# Building the Bridge From Aquatic Nanotoxicology to Safety by Design Silver Nanoparticles

**DOI:** 10.3389/fbioe.2022.836742

**Published:** 2022-03-08

**Authors:** Ilaria Corsi, Martin Federico Desimone, Jimena Cazenave

**Affiliations:** ^1^ Department of Physical, Earth and Environmental Sciences, University of Siena, Siena, Italy; ^2^ Universidad de Buenos Aires, Consejo Nacional de Investigaciones Científicas y Técnicas (CONICET), Instituto de Química y Metabolismo del Fármaco (IQUIMEFA), Facultad de Farmacia y Bioquímica, Buenos Aires, Argentina; ^3^ Laboratorio de Ictiología, Instituto Nacional de Limnología (INALI), CONICET, Universidad Nacional del Litoral, Santa Fe, Argentina

**Keywords:** engineered nanomaterials, nanotoxicology, safety by design, silver nanoparticles, sustainable nanotechnologies, freshwater and marine organisms

## Abstract

Nanotechnologies have rapidly grown, and they are considered the new industrial revolution. However, the augmented production and wide applications of engineered nanomaterials (ENMs) and nanoparticles (NPs) inevitably lead to environmental exposure with consequences on human and environmental health. Engineered nanomaterial and nanoparticle (ENM/P) effects on humans and the environment are complex and largely depend on the interplay between their peculiar properties such as size, shape, coating, surface charge, and degree of agglomeration or aggregation and those of the receiving media/body. These rebounds on ENM/P safety and newly developed concepts such as the *safety by design* are gaining importance in the field of sustainable nanotechnologies. This article aims to review the critical characteristics of the ENM/Ps that need to be addressed in the *safe by design* process to develop ENM/Ps with the ablility to reduce/minimize any potential toxicological risks for living beings associated with their exposure. Specifically, we focused on silver nanoparticles (AgNPs) due to an increasing number of nanoproducts containing AgNPs, as well as an increasing knowledge about these nanomaterials (NMs) and their effects. We review the ecotoxicological effects documented on freshwater and marine species that demonstrate the importance of the relationship between the ENM/P design and their biological outcomes in terms of environmental safety.

## Introduction

### Silver Nanoparticle Market and Application: From Production to Release Into the Environment

Nanomaterials (NMs) were found in structures obtained more than one thousand years ago revealing that they have a long history (Hochella et al., 2019). However, the continued growing interest and development experienced nowadays is mainly due to the ability to design, synthesize, and characterize systems in the nanoscale. In this way, nanoparticles (NPs) are especially designed for applications in a wide range of technologies that affect the chemical, pharmaceutical, electronics, telecommunication, medical, aerospace, automotive, and energy industries, among many others.

Silver nanoparticles (AgNPs) are among the most widely employed and produced. The total world production of AgNPs was estimated to reach thousands of tons per year (Pulit-Prociak and Banach, 2016). The companies participating in the development of AgNPs are distributed in different countries, and the price varies from a few hundred to thousand $/kg (Pulit-Prociak and Banach, 2016). In addition, the offered AgNPs possess different sizes and capping agents. Recent advances in the synthesis, stabilization, and high-scale production of AgNPs have intensified scientific investigation within the nanotechnology field and are the driving force of a new generation of commercial products (Calderón-Jiménez et al., 2017).

Physical and chemical methods were applied to produce AgNPs. These methods offer the possibility to obtain NPs with well-defined size and shape. The most frequent chemical synthesis methods involve bottom-up procedures employing ions as building blocks to obtain AgNPs. The presence of reducing agents allows the formation of metallic silver which subsequently agglomerates to form AgNPs. Classically, silver nitrate is chemically reduced with NaBH_4_ or citrate in aqueous or non-aqueous solvents. The presence of capping agents (i.e., polyvinyl alcohol, PVA) is important to stabilize and prevent the agglomeration of the resulting NPs (Iravani et al., 2014). Among the most widely employed physical methods are laser ablation and evaporation–condensation. Physical methods possess various advantages including the following: *1*) uniformity of NP size, *2*) formation of high amounts, *3*) do not require chemical reagents, and *4*) pure metal AgNP obtention. However, in most cases, these methods are time and energy consuming (Iravani et al., 2014). The synthesis procedure employed for the AgNP production has the potential to greatly influence the global environmental impact. Temizel-Sekeryan and Hicks evaluated the environmental impact of six different AgNP synthesis procedures based on a mass unit of 1 kg of AgNPs. The two chemical methods evaluated were microwave and chemical reduction, while the four physical methods analyzed were flame spray pyrolysis, arc plasma, spark system, and reactive magnetron sputtering. The estimation suggests that the environmental impact of AgNPs is highly influenced by the synthesis procedure, scale, and intended product application (Temizel-Sekeryan and Hicks, 2020). Indeed, on a mass basis, the flame spray pyrolysis physical method generates higher environmental impact. Interestingly, another physical method, the reactive magnetron sputtering, produces the lowest environmental impact. Moreover, the authors conclude that scaling up the production can significantly diminish (90%) the environmental impact of AgNP production. Alternatively, the number of publications in the field of “green synthesis” experienced a sharp increase during the last decade because of employing environment-friendly and sustainable processes (Catalano et al., 2021). Furthermore, plant extracts would work as both green reducing and stabilizing agents. This offers several advantages because it is a one-pot and economic process which leads to AgNPs with antimicrobial and antioxidant properties (Galdopórpora et al., 2021). The latter is due to the adsorption of molecules of the plant extract on the AgNPs. Alternativelly, microorganisms were also employed for the green synthesis of AgNPs. Singh et al. (2015) employed the strain *Brevibacterium frigoritolerans* DC2 for the large-scale production of AgNPs without employing harmful chemicals or energy-consuming physical approaches. The exopolymer secreted by *Ochrobactrum rhizosphaerae* has been employed for the reduction of silver ions and stabilization of the resulting AgNPs (Gahlawat et al., 2016).

AgNPs possess interesting physicochemical properties such as catalytic activity (Xu et al., 2006), high thermal and electrical conductivity (Alshehri et al., 2012; Huang et al., 2019), and surface plasmon resonance (Smitha et al., 2008), which leads to a variety of scientific applications and to the development of new products.

The antimicrobial activity of silver has been known for centuries; thus, it is not surprising that the main application of AgNPs is related to this effect. AgNPs exert activity predominantly through the release of silver ions followed by increased membrane permeability, disruption of DNA replication, and affecting proteins and enzymes (Marambio-Jones and Hoek, 2010). Indeed, AgNPs re-emerged as an effective alternative for the treatment of infections caused by antibiotic-resistant bacteria and viruses (Rai et al., 2009; Huh and Kwon, 2011; Dheyab et al., 2021). This remarkable effect was exploited in various applications in the medicinal field including wound dressings (Municoy et al., 2020; Antezana et al., 2021), medical implant materials (Basova et al., 2021), and coatings on medical devices to reduce nosocomial infection rates (Chaloupka et al., 2010; Catalano et al., 2016).

**FIGURE 1:**
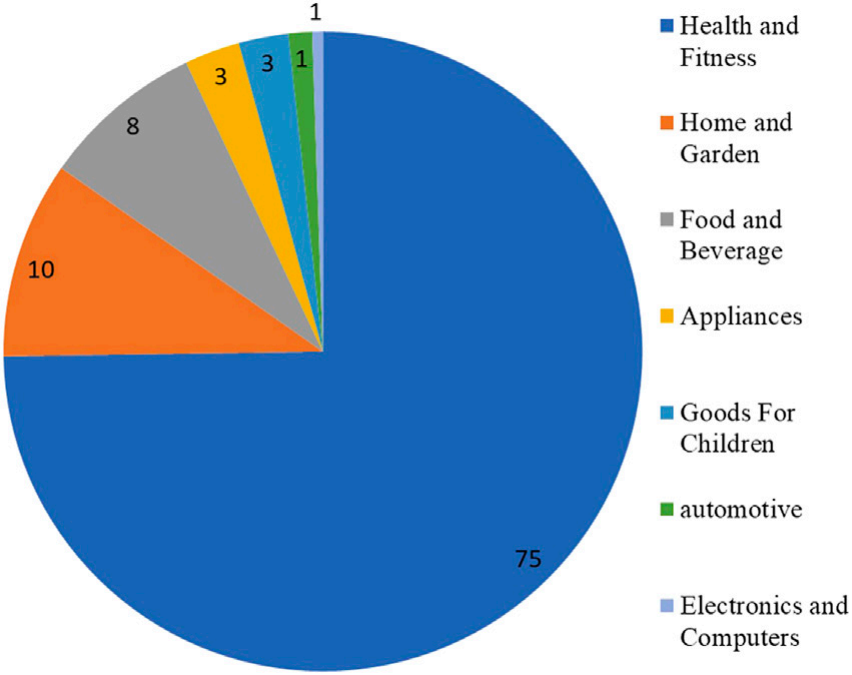
Percentage of products containing AgNPs by categories (source: year 2021, The Nanodatabase, https://nanodb.dk/en/). The Nanodatabase is developed by the DTU Environment, the Danish Ecological Council and Danish Consumer Council.

In parallel, the production of antimicrobial textiles with AgNPs has experienced a great deal of attention (Dastjerdi and Montazer, 2010; Zille et al., 2014). The textile industry took advantage of this growing interest and produced several products which contained AgNPs (i.e., reusable face masks, socks, and uniforms). The resulting textiles possess antimicrobial and antiviral activity; thus, the textile industry has become a very active industry, especially during the coronavirus pandemic. Indeed, silver represents ca. 15% of the products listed in the inventory for products that contain NMs. In this sense, the category with the highest number of products with AgNPs is health and fitness (ca. 75%) followed by home and garden (10%) (Figure 1) (The Nanodatabase, 2021).

Sim et al. (2018) analyzed the expanding applications of silver in medicine, healthcare, and other daily life activities, with a focus on the patents registered. The analysis of patents describing antimicrobial silver applications revealed a six-fold growth in the period 2007–2017, while the most interesting applications described were personal care, domestic, and agricultural and industrial products.

The augmented production and wide range of application of AgNPs inevitably lead to environmental exposure with consequences on human and environmental health. Undoubtedly, the analysis of the fate and effects of AgNPs is of paramount importance since they will eventually enter the waste stream and make their way to a wastewater treatment plant and then into the natural environment. They can reach the environment during usage or disposal of AgNPs or consumer products that contain AgNPs. Actually, different methods are under evaluation for nanowaste management with the aim to diminish their potential release into the environment (Jane Cypriyana et al., 2021).

However, the analysis of septic tank sludge spiked with AgNPs suggests that the effluent and sludge are sources of both AgNPs and Ag^+^ ions to natural waters (Bolaños-Benítez et al., 2020).

Thus, human and environmental safety issues must be addressed due to the increasing exposure possibilities. Recently, AgNP inhalation produced harmful effects and disruption of the alveolar–capillary barrier integrity with increased epithelial permeability along with cell and plasma protein leakage into the alveolar space, which suggests an impaired lung function (Garcés et al., 2021). Indeed, Potter et al. (2019) analyzed AgNP consumer products after contact with human synthetic stomach fluid followed by exposure to wastewater sludge. The rates of conversion of metallic silver to silver sulfide were dependent on the particle size and capping agents. The authors concluded that the transformation of the AgNPs may be somewhat unpredictable in the environment because it is affected by several factors. Moreover, AgNPs release Ag^+^ ions upon dissolution in aqueous media. The dissolution trends of AgNPs in consumer products and ENMs with citrate, polyvinylpyrrolidone (PVP), and polyethyleneimine (PEI) coatings further confirm that differences would be attributed to capping agents, particle size, and total AgNP surface area (Radwan et al., 2019). Furthermore, Ag^+^ release from AgNPs decreased 28% when humic acids were present in the media, highlighting that organic matter has an important impact on NP stability (Ale et al., 2021b).

## Environmental Behavior of Silver Nanoparticles

**FIGURE 2:**
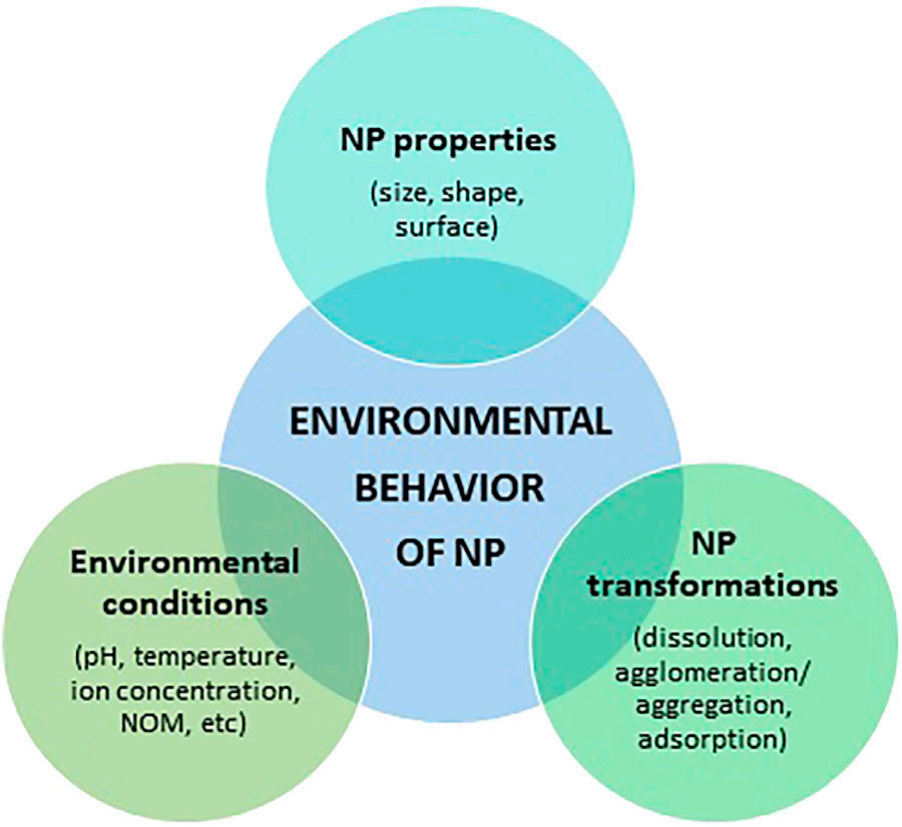
Factors influencing the environmental behavior of AgNPs.

The environmental behavior of AgNPs depends on NP physical properties, NP environmental transformations, and the influence of environmental conditions (Figure 2). In natural aquatic systems, all these fluctuating factors act simultaneously, which means that the transport, behaviour, and fate of AgNPs are complex and difficult to predict. Despite this, understanding these transformation processes is vital for the assessment of the environmental risks of AgNPs (Zhang et al., 2018). It is also important to mention that the behaviour and transformations of AgNPs have been previously reviewed by other authors (Fabrega et al., 2011; Zhang et al., 2016, Zhang et al., 2018; Shevlin et al., 2018; Jorge de Souza et al., 2019; Xu et al., 2020)

### Effects of NP Physical Properties

AgNPs can reach the aquatic environment by various routes and exceed ecotoxicity thresholds for aquatic species (Syafiuddin et al., 2017). The average concentration of AgNPs in treated urban waste waters has been estimated in the range between µg and ng per litre (Li et al., 2016), and more recent findings demonstrated a high removal efficiency by wastewater treatment between 85 and 97% on a case-by-case analysis (Cervantes-Avilés et al., 2019; Nabi et al., 2021). AgNPs of the smallest size (<200 nm) are more likely to escape the wastewater treatment, thus ending up into effluents and even somehow further reducing their dimension down to 50 nm (Cervantes-Avilés et al., 2019; Nabi et al., 2021).

Ecotoxicity of AgNPs has been associated with the smallest sizes (below 20 nm); therefore, the aquatic ecological risks are also dictated by the size of AgNPs released and/or transformed in the final receiving water bodies (Nel et al., 2006; Fabrega et al., 2011; Ivask et al., 2014).

Particle size is also an important factor that affects the kinetics of AgNP dissolution, with a smaller size having higher specific surface areas and more surface-active sites for Ag^+^ production than larger AgNPs (Levard et al., 2012; Behra et al., 2013; Zhang et al., 2016, Zhang et al., 2018). Studies of AgNP dissolution showed that particle size or surface coating can affect its long-term stability in freshwater environments (Zhang et al., 2016). For instance, Dobias and Bernier-Latmani evaluated the AgNP dissolution of different sizes (5, 10, and 50 nm) and capping agents such as polyvinylpyrrolidone (PVP), tannic acid (Tan), and citrate (Cit) exposed to river and lake water for up to 4 months (Dobias and Bernier-Latmani, 2013). The results showed that small AgNPs (5 nm, PVP and Tan) are dissolved rapidly and almost completely, while larger AgNPs (50 nm) have the potential to persist for an extended period of time. In addition, the authors observed that PVP- and Tan-AgNPs were more prone to Ag^+^ release than Cit-AgNPs.

Several attempts have been made in the understanding of the dissolution drivers of AgNPs such as, for instance, by functionalization of the particle surface which resulted in a highly variable degree of particle dissolution and low ecotoxicity (Gondikas et al., 2012; Levard et al., 2013; Schiavo et al., 2017; Wu et al., 2017; Pem et al., 2019).

### Environmental NP Transformations and Influence of Environment Conditions

Once released into aquatic environments, AgNPs are subject to several physical, chemical, and biological transformation processes, such as dissolution to Ag^+^, adsorption to macromolecules or cells, and aggregation or agglomeration. These transformations, in turn, are dependent on intrinsic NP properties (e.g., size, capping, and surface charge) and the environmental conditions of the surrounding medium into which they were released (e.g., temperature, pH, ionic strength, the availability of ligands, and light) (Vale et al., 2016; Rearick et al., 2018).

The interplay of either bare AgNPs and/or Ag-based nano-enabled products (NEPs) with physico-chemical and biological variables of the receiving water bodies (e.g. pH, temperature, and natural organic matter) will dictate the key transformation processes which NPs undergo such as, for instance, homo/heteroaggregation, agglomeration, ions dissolution, and complexation, which will affect exposure concentration scenarios, bioavailability, and ecotoxicity (Baalousha et al., 2018; Corsi et al., 2020; Corsi et al., 2021). This is true for the majority of ENM/Ps, and in the case of silver, dissolution is still considered a main driver of aquatic ecotoxicity of AgNPs (Petosa et al., 2010; Dong et al., 2017).

The dissolution process is also affected by specific intrinsic properties of the particle itself (e.g., surface coating) and the physico-chemical characteristics of the receiving water media, for instance, osmolarity and natural organic matter (NOM) (McLaughlin and Bonzongo, 2012; Asadi Dokht Lish et al., 2019; Prosposito et al., 2019). Although the dissolution of silver ions (Ag^+^) in water has been reported to increase with ionic strength, the abundance of chloride species (Cl^−^) favours also the Ag complexation and precipitation, thus making it less bioavailable for exposed aquatic species (Li et al., 2020).

Odzak et al. (2015) examined the dissolution of AgNPs over a period of 9 days in different types of natural waters as wastewaters from treatment plants (WWTP) and four lakes. The results of this study indicated that AgNP dissolution was favoured at low ionic strength and low pH.

Under simulated freshwater environmental conditions, Walters et al., reported higher dissolution rates of AgNPs with increased temperature. Similarly, a sudden flood event also promoted Ag release (Walters et al., 2013). Thus, this study showed that AgNP dissolution is induced by changes in temperature and hydrological level, which might present a greater risk for aquatic organisms.

On the other hand, Zou et al. studied the roles of NOM and dissolved oxygen on AgNP dissolution in natural freshwaters (Zou et al., 2017). In the presence of oxic conditions, a higher degree of dissolution of AgNPs was observed in comparison to anoxic conditions. This could be explained by the fact that dissolution requires the presence of oxidant oxygen. So, if an insufficient supply of oxygen is present, only a very limited AgNP dissolution could occur. Interestingly, in the same study, a notable decrease in the concentration of Ag^+^ was observed after the addition of NOM. This decrease in AgNP dissolution could be explained by several mechanisms, such as the surface adsorption of NOM to block AgNP oxidation sites and/or a reversible reaction of released Ag^+^ to Ag0 with humic/fulvic acids as reductants (Liu and Hurt, 2010). Besides NOM, AgNPs or released Ag ions can react strongly with other ligands (e.g., sulphide and chloride), which will affect their transport and bioavailability in the environment. In freshwaters, sulfidation and chlorination seem to be the most relevant processes through which AgNPs will be transformed into Ag_2_S and AgCl, decreasing the dissolved bioavailable Ag^+^ ions (Levard et al., 2012; Behra et al., 2013). Then, because of their low water solubility, the precipitation of Ag_2_S and AgCl is expected. However, these transformations will depend on some environmental conditions. For example, sulfidation occurs almost exclusively under anaerobic conditions such as those found in wastewater treatment plants (Kaegi et al., 2011; Kent et al., 2014).

As a counterpart, NOM has been demonstrated to slow down the dissolution process *via* the formation of clusters and bridging effects (Wang et al., 2014; Gunsolus et al., 2015). Being prevalent in river and brackish waters, NOM is considered the main driver in limiting AgNP dissolution, while the high ionic strength and algal exudates have a leading role in salt waters.

The adsorption of biomolecules has been shown to either improve NP colloidal stability by acting as an electrostatic barrier which limits agglomeration and aggregation processes or destabilizes dispersed suspensions by forming bridges and saturating surface charges (Surette and Nason, 2016, Surette and Nason, 2019). AgNPs can adsorb different macromolecules present in natural waters (e.g., NOM, extracellular polymeric substances, EPS, and proteins), forming an environmental corona known as *eco-corona*, an external layer acquired by the NPs once released in natural media which affect how they interact with biological systems (Corsi et al., 2020, and reference within; Barbero et al., 2021). *Eco-corona* dictates nanoparticle biological interactions (nano-bio-interactions) which are played by proteins, carbohydrates, and metabolites including nucleic acids that are readily adsorbed on the surface of NPs once in contact with biological entities and their milieu (Chetwynd and Lynch, 2020, and reference within; Wheeler et al., 2021). Formerly described as *protein corona* originating from the adsorption of a protein layer upon contact with human blood, it represents the outmost contact point between the NP and the biological membrane more recently deeper investigated in having a role in NP uptake, disposition, and ultimate nano(eco)toxicity (Lynch et al., 2007, Lynch et al., 2014; Grunér et al., 2016). Both biotechnology and nanotoxicology have already incorporated different aspects of the biomolecular coronas, while in nanoecotoxicology, few attempts have been made by systematic characterization of their composition upon contact with natural water media and biological fluids (Canesi et al., 2017; Grassi et al., 2019, 2020; Nasser et al., 2020; Wheeler et al., 2021, and reference within).

Xu et al. reviewed the role of eco-coronas in determining the effects of ENP and concluded that, in most cases, NOM and EPS coronas were able to alleviate the bioavailability and biological effects on aquatic organisms (Xu et al., 2020). This is in line with another literature review that evidenced that the presence of NOM reduced ENM/P ecotoxicity in 80% of the analysed studies (Arvidsson et al., 2020). In particular, ecotoxicity values (EC_50_, LC_50_, and IC_50_) obtained in the experiments with NOM (at an environmentally realistic concentration range of 0.1–10 mgL^-1^) tended to be 1 to 10 times higher than those without NOM. On the other hand, the increase of EPS in algal cultures (*Chlamydomonas reinhardtii* and *Chlorella vulgaris*) mediated the inactivation of AgNPs and Ag^+^ ions (Stevenson et al., 2013; Zheng et al., 2019). The mitigation of AgNP toxic effects can be attributed to the fact that the formation of a corona could greatly affect the NP characteristics (size), induce aggregation, and result in precipitation. However, the interactions of macromolecules with AgNPs and Ag^+^ ions are still poorly understood, and further studies are needed to elucidate the dynamics of corona formation as well as the mechanisms of stabilization and aggregation of these particles in the aquatic environments.

Particle aggregation determines the sedimentation rate and the mobility of ENM/Ps in the environment. The aggregation and sedimentation of AgNPs can be reduced by the formation of an NOM coating that provides electric and steric stabilization (Cumberland and Lead, 2009; Grillo et al., 2015). However, the aggregative behaviour of AgNPs might be influenced by both the concentration and chemical composition of NOM (Millour et al., 2021). On the other hand, other environmental factors, such as ionic strength and ions (e.g., Cl^−^, SO_4_^2^, Ca^2+^), can favour the formation of the aggregates. Typically, in a freshwater environment, AgNPs remain stable even under low ionic strength (Bathi et al., 2021). However, the increase of ionic strength (for instance, from crystalline rock areas to carbonate rock areas or/and estuarine and marine waters) can significantly raise the aggregation of AgNPs (Behra et al., 2013; Zhang et al., 2016). Some studies reported that as ionic strength increased, AgNPs were more unstable and more aggregates were formed (Chambers et al., 2014; Yue et al., 2015). In another study, the addition of sodium and calcium at low ionic strengths (relevant to freshwater and estuarine systems) increased the aggregation of Cit-AgNPs (Cumberland and Lead, 2009).

In this sense, although the increased NP size due to agglomeration is generally related to lower toxicity (Tortella et al., 2020), the degree of particle agglomeration in the three exposure media positively correlated with the toxicity of Cit-AgNPs on gill cell viability (Yue et al., 2015). One explanation might be that agglomeration and deposition could enhance the interaction of AgNPs with gill cells attached to the bottom of wells in the *in vitro* exposure system. However, it is also important to mention that the exposure medium which better reflected the freshwater environment (low ionic strength) supported cell survival and stabilized the AgNPs for at least 24 h.

The mode of action (MoA) of AgNPs towards biota and, in particular, on single-cell organisms is still uncertain based on low repeatability and reproducibility of ecotoxicity data on microorganisms, algae, and cell lines. The dissolution of silver ions by AgNPs has been recognized as one MoA, thus causing similar biological effects as those of bulk Ag; however, recent findings revealed that nanoscale Ag can penetrate the cell membrane and generate oxidative stress by the release of free radicals even under limited dissolution (Batchelor-McAuley et al., 2014; Völker et al., 2015; Malysheva et al., 2016; Kędziora et al., 2018; Kleiven et al., 2019). Therefore, ecotoxicity either mediated by Ag ion dissolution or by AgNPs must be taken into consideration and investigated on a case-by-case analysis based on specific properties of the AgNPs tested. As detailed above, similarly for all ENM/Ps, AgNPs will undergo significant transformations when released into the natural environment and finally acquire new properties driven by the initial feature of the NP and those of the receiving water media (e.g., freshwater vs brackish or saline), for instance, NOM ionic strength, temperature, and pH (Corsi et al., 2020). Therefore, MoA of AgNPs towards aquatic biota leading to ecotoxicity is, thus, still far from being fully understood (Lead et al., 2018). The current gap between predicted environmental concentrations (PECs ≤ μg L^−1^) and effect concentrations (range mgL^−1^-µgL^−1^) for aquatic species is expected to be significantly reduced in the near future due a recent increase of AgNP use in textiles and sanitizing products, with the latter being a consequence of the COVID-19 worldwide pandemic (Hamouda et al., 2021; Valdez-Salas et al., 2021).

Nano-ecotoxicology has made several progresses in the last years by identifying those factors able to cause changes in ENM/Ps and NP peculiar properties when released in the aquatic environment (Maurer-Jones et al., 2013; Lazareva and Keller, 2014; Corsi et al., 2021). Preliminary findings have unraveled various exposure scenarios according to the peculiar properties of the receiving water media (freshwater, brackish, or saline) which has now become a requirement for ecological risk assessment and for conducting standardized protocols for ecotoxicity testing (OECD, 2020a). Furthermore, up to now, nano-ecotoxicity studies have been performed using pristine ENM/Ps rather than commercial formulations as NEPs, with the latter being mostly likely to end up into water bodies across their lifecycle (Mitrano et al., 2015; Pourzahedi et al., 2017; Salieri et al., 2018; Potter et al., 2019). In particular, NEPs in liquid formulations could more easily dissolve NPs in water and reach aquatic organisms (Vílchez et al., 2016; Nowack and Mitrano, 2018).

**FIGURE 3:**
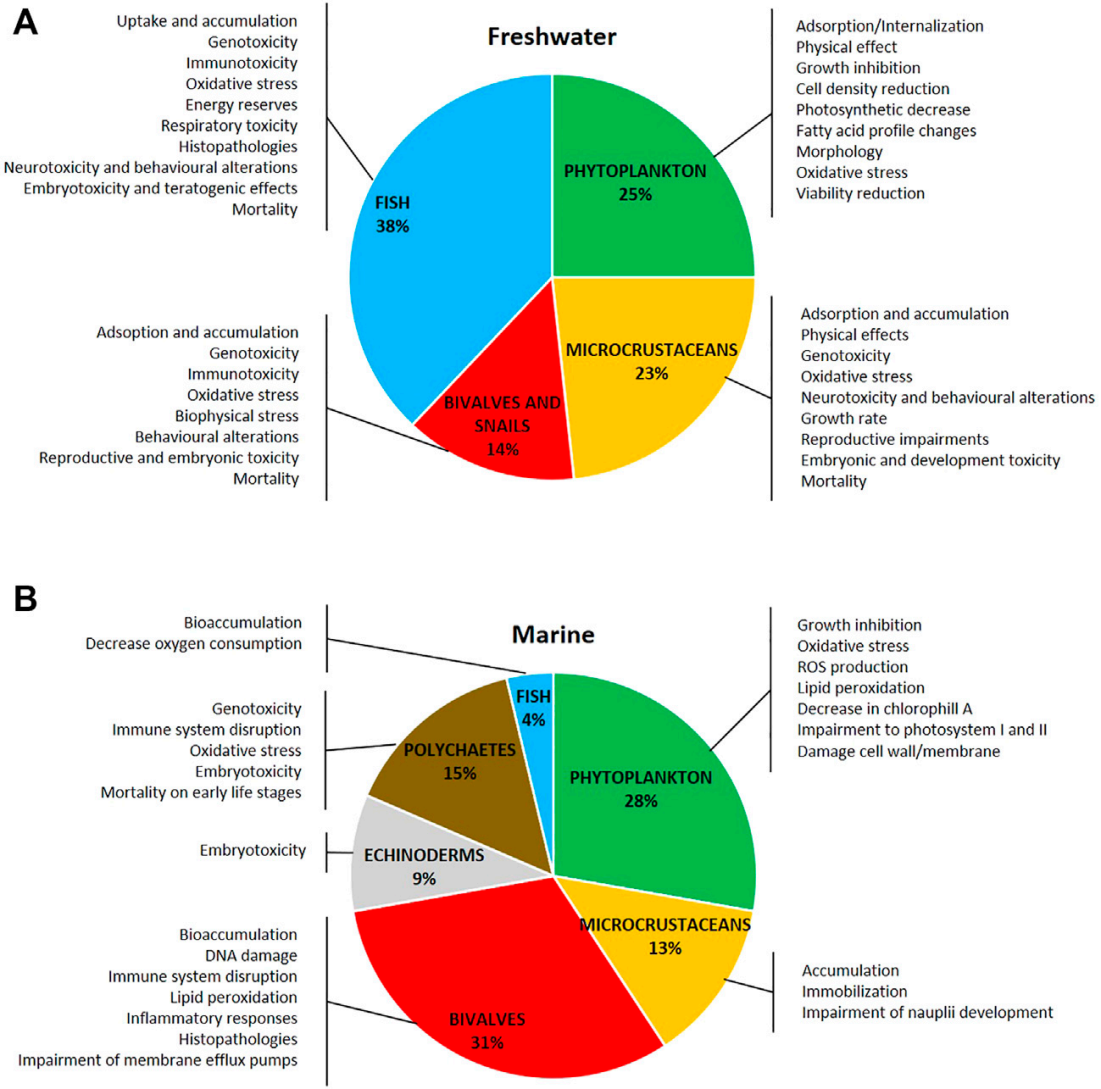
Distribution of taxa investigated and the main toxic effects of silver nanoparticles on **(A)** freshwater and **(B)** marine organisms. The literature used refers to research articles cited in the text (freshwater: 116 articles; marine: 54 articles).

The ecotoxicity of AgNPs in freshwater and marine species has been reviewed and commented based on NP properties (size, shape, and surface coatings), and their behavior in water media and ultimate reported biological outcomes including dissolution when observed and the bioaccumulation of silver ions and adsorption on the body surface have been reported. Ecotoxicity related to Ag ENM/NP dissolution or to a specific effect has been reported and discussed. Figures 3A,B show a schematic representation of the distribution of taxa investigated in ecotoxicity studies in (A) freshwater and (B) marine species and related documented effects.

## Aquatic Ecotoxicity of Silver Nanoparticles

### Freshwater Environment

#### Phytoplankton

Nowadays, understanding the pathways and mechanisms of AgNP toxicity on phytoplankton has become essential. Microalgae are the primary producers of food webs, and any damage to their function may impact at higher trophic levels and/or the whole ecosystem. The NP internationalization and toxicity of algae have been addressed in several recent reviews (Chen et al., 2019; Wang et al., 2019; Nguyen et al., 2020; Mahana et al., 2021). Despite the extensive research, the toxic mechanism of AgNPs in freshwater algae is still a matter of debate.

Several studies support the hypothesis that AgNPs are not internalized by algae but absorb onto the cell surface and suggest that only Ag ions can penetrate the cells (Piccapietra et al., 2012; Leclerc and Wilkinson, 2014; Li et al., 2015; Ribeiro et al., 2015; Yue et al., 2017). In *Chlamydomonas reinhardtii*, analyses from darkfield microscopy coupled with hyperspectral imagery indicated that only Ag^+^ were crossing the algal membrane and that the presence of silver inside the cells was more likely due to Ag^+^ reduction than to AgNPs internalization (Leclerc and Wilkinson, 2014). Moreover, in a cell wall-free mutant of *C. reinhardtii*, no AgNP uptake was detected, indicating that not only the cell wall of algae but also the cell membrane constitutes a barrier for particle internalization (Piccapietra et al., 2012). Similarly, other studies concluded that AgNPs are not internalized by *Euglena gracilis* (a green alga having no cell wall) but adsorb onto the algae cell surface (Yue et al., 2017) and to the pellicle (Li et al., 2015). Furthermore, some authors have also observed that the interaction between freshwater algae and AgNPs led to the formation of large aggregates (Oukarroum et al., 2012; Zouzelka et al., 2016), making it difficult to distinguish whether AgNPs are located outside or inside of cells using optical microscopy. By using the Coherent Anti-stokes Raman Scattering (CARS) microscope, large aggregates of AgNPs on the external surface of *Raphidocelis subcapitata* were observed, with no evidence of NP internalization into the algal cells (Ribeiro et al., 2015). Thus, the authors highlighted that AgNP toxicity might be mediated by Ag^+^ internalization or/and through a physical effect induced by NP agglomerate. For instance, depending on their size, agglomerates may interfere with the algae growth and photosynthesis and/or lead to faster sedimentation of cells (Ribeiro et al., 2015).

On the contrary, a limited number of studies suggested that AgNPs can be taken in and accumulated inside the algal cells, where they exert their toxic effects (Miao et al., 2010; Kalman et al., 2015; Wang et al., 2016). In an experiment, the freshwater alga *Ochromonas danica* was exposed to AgNPs, and glutathione (GSH) was also added to eliminate the indirect effect of Ag^+^ (Miao et al., 2010). The results demonstrated that the presence of GSH remarkably reduced free Ag^+^ concentration in the media, reaching levels even lower than the non-observed effect concentration of Ag^+^ itself. However, the toxic effect was still exerted indicating that AgNPs could enter algal cells. On the other hand, transmission electron microscopy (TEM) images revealed AgNPs localised in starch granules within the chloroplast of *Chlorella vulgaris* (Kalman et al., 2015). This finding demonstrated the intracellular uptake and suggested that granules may act as a storage site for NPs in this alga (Kalman et al., 2015). By using a combination of high-resolution imaging and *in situ* detection spectroscopic techniques, Wang et al. (2016) identified the existence of Ag particles with a diameter of 10–20 nm inside *C. reinhardtii* cells. These observations suggested that AgNPs would enter the periplasmic space after cellular internalization into the alga.

Although AgNP internalization is still controversial, some possible NP uptake pathways have been hypothesized, such as endocytosis, across porous cell walls (in the case of NPs less than 20 nm in size), through ion channels and undetermined carriers (Oukarroum et al., 2012; Behra et al., 2013; Yue et al., 2017; Zheng et al., 2019; Nguyen et al., 2020, among others).

Whatever the interaction between AgNPs and algae cells (direct particle–cell surface attachment, internalization through the cell membrane, release of Ag^+^ through dissolution, or some combination of these), scientific literature has evidenced that AgNPs exert toxicity on freshwater microalgae. Overall, the exposure to AgNPs inhibited microalgal growth (Miao et al., 2010; Burchardt et al., 2012; Becaro et al., 2015; Kleiven et al., 2019; Romero et al., 2020; Qu et al., 2021), reduced algae cell density (Sendra et al., 2017; Khoshnamvand et al., 2020), decreased the photosynthetic activity and the chlorophyll content (Navarro et al., 2008; Oukarroum et al., 2012; Książyk et al., 2015; Nguyen et al., 2018), caused changes in fatty acid profile (Behzadi Tayemeh et al., 2020), affected the morphology of algae cells (Li et al., 2015; Romero et al., 2020), increased reactive oxygen species (ROS) intracellular content and generated oxidative stress (Oukarroum et al., 2012; Qian et al., 2016; Sendra et al., 2017; Nguyen et al., 2018), damaged membrane integrity, and also, reduced cell viability (Oukarroum et al., 2012; Taylor et al., 2016; Sendra et al., 2017; Nguyen et al., 2018). Moreover, recent studies have intended to shed some light on these toxic mechanisms, based on metabolomics and proteomics analyses (Zhang et al., 2020b; Qu et al., 2021).

However, the biological effects mainly depend on intrinsic ENM/P characteristics, such as size, shape, surface coating, and electrical properties. For example, the toxicity of AgNPs decreases with increasing primary particle size. After a 72 h growth inhibition assay, the EC_50_ values (concentration at which a 50% inhibition of the growth rate is observed) of five different sized AgNPs (from 10 to 80 nm) for *Pseudokirchneriella subcapitata* confirmed that rule (Table 1) (Ivask et al., 2014). The results obtained by Zouzelka et al*.* (2016) showed that AgNPs exhibited substantial toxicity towards Chlorococcales and filamentous algae, which mostly correlated with their particle size. Thus, the highest toxicity was also found for the smallest particles (5 nm) in comparison to larger ones (37–100 nm).

**TABLE 1:** Comparison among EC_50_ and LC_50_ values calculated for AgNPs in freshwater and marine taxa.

Taxa	Freshwater			Marine		
	*Species*	*EC*_*50*_ */LC*_*50*_	*References*	*Species*	*EC*_*50*_ */LC*_*50*_	*References*
Phytoplankton	*Pseudokirchneriella subcapitata = Raphidocelis subcapitata*	0.18–1.14 mgL^−1^	Ivask et al. (2014), Sohn et al. (2015)	*Talassiosira* sp*.*	107.21 ± 7.43 µgL^−1^	Pham (2019)
		0.74 mgL^−1^ (spheres)				
		2.573 mgL^−1^ (AgNW)				
	*Chlorella vulgaris*	9.2 mgL^−1^ (Cit)	Kalman et al. (2015)	*Phaedactylum tricornutum*	143–184 µgL^-1^	Sendra et al. (2017)
		9.3 mgL^−1^ (PVP)				
		49.3 mgL^−1^ (PEG)				
Microcrustaceans	*Daphnia magna*	1->200 μgL^1^	Allen et al. (2010), Li et al. (2010), Poynton et al. (2012), Ivask et al. (2014), Mackevica et al. (2015), Conine et al. (2017)	*Artemia salina*	5.5 × 100 µgL^−1^ (PVP)	Arulvasu et al. (2014), Becaro et al. (2015), Gambardella et al. (2015), An et al. (2019), Asadi Dokht Lish et al. (2019)
		1.8 µgL^1^ (Cit)			10.70 ± 1.3 mgL^−1^ (spheres)	
		10.6 µgL^1^ (PVP)			0.43 ± 0.04 mgL^−1^ (AgNW)	
					*LC*_*50*_ (24 h) 9.96 (6.64–14.94) mgL^−1^	
					*LC*_*50*_ (48 h) 3.79 (2.28–6.29) mgL^−1^	
					*EC*_*50*_ (24 h) 3.56 (1.99e6.35) mgL^−1^	
					21.35 ± 5.67 mgL^−1^ (Cit)	
					42.44 ± 11.30 mgL^−1^(Cit)	
	*Daphnia dentifera*	34–292 µgL^1^	Conine et al. (2017)	—	—	—
Bivalves and snails	*Physa acuta*	2.18 µgL^−1^ (without sediment)	Bernot and Brandenburg, (2013)	*Mytilus galloprovincialis*	2.05 mg L^−1^ (PVP/PEI) (hemocytes), 4.74–9.5/4.39–8.69 mgL^−1^ (Mal-20, 40,100) (hemocytes/gills), 19.13–22.79/18.22–20.45 mgL^−1^ (spheres 20–80) (hemocytes/gills)	Katsumiti et al. (2015), Duroudier et al. (2019b)
		10 µgL^−1^ (with sediment)				
Benthic-grazers	—	—	—	*Paracentrotus lividus*	3 mgL^−1^ (embryos)	Šiller et al., 2013, Gambardella et al. (2015)
					0.55 mgL^−1^ (sperm motility)	
Fish	*Danio rerio*	1.22 mgL^−1^ (Cit-20)	Abramenko et al. (2018), Caceres-Velez et al. (2018), Caceres-Velez et al. (2019), Liu et al. (2019)	—	—	—
		2.14 mgL^−1^ (Cit-100)				
		1.34 mgL^−1^ (PVP-20)				
		2.57 mgL^−1^ (PVP-100)				
		0.0169 mgL^−1^ (AgPL)				
		0.0415 mgL^−1^ (spheres)				
		1.19 mgL^−1^ (embryos without HA)				
		3.56 mgL^−1^ (embryos with HA)				
		25.0 mgL^−1^ (adults without HA)				
		40.56 mgL^−1^ (adults with HA)				
	*Oryzias latipes*	1.8 mgL^−1^ (spheres)	Abramenko et al. (2018)	—	—	—
		4.18 mgL^−1^ (AgNW)				

Concerning the shape of the ENM/Ps, the 72 h EC_50_ of silver nanospheres for *Raphidocelis subcapitata* (= *P. subcapitata*) was about 3.5-fold lower than the corresponding values of silver nanowires (AgNWs) (Table 1) (Sohn et al., 2015). In parallel, the authors reported a greater amount of Ag^+^ released from the nanosphere surface in comparison with AgNWs, which explained the greater toxicity displayed by nanospheres. Thus, the greater toxicity seemed to be related to the amount of Ag^+^ released rather than the ENM’s shape. In another recent study, Nam and An (2019) compared the effects of nanospheres (40 nm diameter), AgNWs (21,000 nm length × 42 nm diameter), and Ag nanoplates (AgPL, 57 nm diameter) on growth and photosynthetic performance of the green alga *Chlorococcum infusionum*. The results showed toxicity in the order AgPL > AgNW > Ag nanospheres.

Another factor influencing ENM/P toxicity is surface coating. Kalman et al. (2015) investigated the toxicity of silver nitrate (AgNO_3_) and AgNPs coated with PVP, polyethylene glycol (PEG), and Cit to *C. vulgaris*. The highest growth rate inhibition after 72 h was found for dissolved silver (IC_50_ of 5.3 mgL^−1^), followed by PVP and Cit-coated AgNP treatments, which exhibited similar toxicity, whereas PEG-AgNPs displayed the lowest inhibitory effects to the algae (Table 1). On the other hand, Navarro et al. (2015) assessed the effects of AgNO_3_ and nine differently coated AgNPs on the photosynthesis of *C. reinhardtii*. Although all AgNPs were differentially toxic to the photosynthetic yield of algae (for example, dexpanthenol-, PEG-, and PVP-coated NPs were strongly toxic, while Cit, chitosan, or carbonate were less toxic), the authors demonstrated the predominant toxicity role of Ag^+^. Thus, it was suggested that coatings may be modulating Ag^+^ release and, consequently, nanoparticle toxicity.

Likewise, surface charge mediates uptake and internalization processes and, thus, strongly affects AgNP toxicity. Zhang et al. (2020b) revealed the effects of surface charge on the bioaccumulation dynamics of AgNPs in *C. vulgaris*. The results showed that positive AgNPs had almost 20 times greater uptake rate than negative ones. This could be explained by the electrostatic attraction between the negatively charged alga cell membrane and the positive AgNPs. On the contrary, negative AgNPs have a low affinity for the anionic cell membrane, which would reduce the adsorption or internalization of NPs by algal cells. Additionally, Zhang et al. (2020a) analysed the toxic mechanisms of AgNPs with different surface electrical charges in *C. vulgaris* using a proteomics approach. The positively charged AgNPs interfered with pathways related to protein synthesis and DNA genetic information transmission. Meanwhile, the negative AgNPs particularly affected oxidative stress phosphorylation pathways and those related to amino acid synthesis.

The toxic effects of ENM/Ps can also be influenced by certain environmental conditions (e.g., pH, ionic strength and dissolved organic matter). Among them, the dissolved organic matter (DOM) has been demonstrated to mitigate the toxicity of biologically synthesized AgNPs to *C. vulgaris* (Khoshnamvand et al., 2020). Specifically, AgNPs decreased the biomass and chlorophyll-a content in a dose-dependent manner, enhanced algal aggregation, and decreased cell size, especially at higher concentrations. However, the presence of DOM in the media prevented cellular aggregation and size reduction of algal cells.

Another possible mechanism that may mitigate AgNP toxicity is the production of EPS from algal cells. After EPS was extracted from *C. vulgaris*, a remarkable increase of total accumulated Ag content for both AgNP and dissolved ion (from AgNO_3_) treatments was observed, with this effect being even greater on NPs than Ag^+^ (Zheng et al., 2019). These findings indicated that EPS displayed barrier effects on AgNPs.

#### Microcrustaceans

Based on the literature review of the toxic effects of AgNPs in freshwater microcrustaceans, we found that the majority of the studies have been performed with the cladoceran *Daphnia magna*, a common and standardized species for acute and chronic ecotoxicity testing (Baun et al., 2008; Gutierrez et al., 2021). This is due to its easy availability, rapid reproduction, short lifespan, and sensitivity. Thus, information about other cladoceran species is scarce, and the interspecific variation of sensitivity in microcrustaceans has rarely been investigated (Völker et al., 2013; Sakamoto et al., 2015; Cui et al., 2017).

The daphnids are an important link between the primary producers (algae) and secondary consumers (fish). Daphnids can filter relatively large volumes of water and consume algae, bacteria, and organic and inorganic particles. Based on this, the AgNP uptake may occur through different routes, including surrounding media and diet. Zhao and Wang (2010) demonstrated that radiolabelled AgNPs were more efficiently assimilated by *D. magna* upon dietary exposure, when compared to waterborne exposure. This finding highlights the importance of AgNP transport along the food chain. However, assuming that a combination of both waterborne and dietary exposures is what most likely occurs in natural environments, Ribeiro et al. (2017) compared the potential of *D. magna* to accumulate Ag from AgNPs through different exposure routes (water, diet, and combined water+diet). The authors found that simultaneous waterborne and dietary exposures to AgNPs induced greater Ag concentrations in *D. magna*, with water uptake being the major contributor.

Asghari et al. (2012) observed the accumulation and adsorption of AgNPs in the gut, under the carapace, on the external body surface and in the appendages of *D. magna*. In general, the accumulation of ENM/Ps occurs when the elimination rates are lower than the uptake rates (Turan et al., 2019). In this sense, Borase et al. (2019) found that AgNPs accumulated in the gut of *Moina macropopa* as the formed nanoaggregates make its excretion difficult, thereby resulting in an increased metal body burden. Thus, AgNP uptake and accumulation may pose a risk to higher trophic levels in the food web and may be a reason for triggering toxic effects on microcrustaceans themselves (Turan et al., 2019).

The acute toxicity (Lethal Concentrations50, LC50) of AgNPs to microcrustaceans is extremely variable ranging from a few micrograms per litre (as reported by Allen et al. (2010) and Li et al. (2010) to values 100-fold higher (Mackevica et al., 2015; Conine et al., 2017) (Table 1). The differences among LC_50_ values are related to particle characteristics (such as size, capping agents, and surface chemistry), experimental conditions (i.e., test media composition, exposure time, and with or without food), and test species (age and uptake strategy).

With regard to sublethal effects, several endpoints have been evaluated in freshwater microcrustaceans. Some studies have demonstrated that AgNPs can induce genotoxicity. Specifically, an increase in DNA strand breaks (Park and Choi, 2010) and distinct gene expression profiles have been reported in *D. magna* exposed to nanospheres and AgNWs (Poynton et al., 2012; Scanlan et al., 2013; Hou et al., 2017). Although transcriptome analysis revealed some molecular mechanisms of ion binding and several altered metabolic pathways, further studies are necessary to elucidate the molecular mechanisms underlying AgNP toxicity in different microcrustacean species. In addition, at a biochemical level, enzymes associated with neurotransmission, oxidative stress, and metabolism were affected by AgNPs in *D. magna* and *M. macrocopa* (Ulm et al., 2015; Borase et al., 2019; Galhano et al., 2020).

In addittion, AgNPs disturb normal behaviour in microcrustaceans. Asghari et al. (2012) observed erratic swimming in the early stages of *D. magna*, whereas in the late stages and high AgNP concentrations, they often migrated to the water surface or the bottom. A similar trend for changing allocation time was also reported by Galhano et al. (2020), who observed that animals exposed to AgNPs spent more time at the bottom and in the middle than at the top. These behavioural changes might be related to the adsorption of AgNPs to the animal body, to an inhibitory effect of the neurotransmission enzyme AChE, or to immobilization effects (at high concentrations), among other mechanisms.

Other ways by which ENM/Ps exhibit negative effects on microcrustaceans are growth retardation and reproductive impairments. Such endpoints are determined after chronic (21 days) and multi-generation exposure studies. For instance, the exposure to environmentally relevant concentrations of AgNPs caused a reduction in the mean number of offspring in six generations of *D. magna* (Hartmann et al., 2019). During chronic toxicity testing, cladocerans (*Daphnia* spp.) and rotifers (*Brachionus calyciflorus*) exposed to AgNPs showed significant inhibition on the population growth rate (Zhao and Wang, 2011; Völker et al., 2013; Martins et al., 2020). Neonates from pre-exposed parental daphnids did not completely recover when transferred into clean water. On the contrary, the population growth rate of rotifers was recovered across five generations, although a delay in the reproduction was observed, probably indicating that the animal fitness was affected (Martins et al., 2020). Furthermore, Park et al. (2021) found that AgNPs not only could lead to negative effects on the reproductive success of *D. magna* over two consecutive generations but also cause several embryonic developmental arrests and abnormalities. These findings support the idea that effects caused by AgNMs may persist in microcrustaceans with time with risks at the population level.

Several studies highlighted the importance of size on AgNP toxicity in microcrustaceans, where smaller NPs are more toxic than the larger ones. Smaller particle size offers a greater specific surface area which increases the rate of Ag^+^ release. For instance, exposure to smaller AgNPs (20–40 nm) caused higher acute toxicity to *D. magna* than their larger counterparts (60–110 nm) (Seitz et al., 2015; Hou et al., 2017). Similarly, the immobilisation of *D. magna* neonates increased with decreasing particle size (10 > 20 > 40 > 60 > 80 nm) (Ivask et al., 2014), and the influx rates of AgNPs into daphnids were also size dependent (20 > 50 > 100 nm) (Zhao and Wang, 2012). In the same way, Scanlan et al. (2013) reported that short-length AgNWs were more toxic than long-length AgNWs against *D. magna*. On the contrary, Cui et al. (2017) found that AgNWs of 20 μm were more toxic than those of 10 μm in *D. galeata* and *D. magna*.

The shape is another characteristic that may affect nanotoxicity in microcrustaceans. For example, a study by Sohn et al. (2015) reported that spherical AgNPs (diameter 5–25 nm) were more toxic (immobilization) than AgNWs (length 10 ± 5 μm) to neonate *D. magna*. This is probably due to the higher amount of Ag ions released from the surface of AgNPs compared with AgNWs. Additionally, the authors observed abnormal swimming, AgNM ingestion, brood chamber pigmentation, and small bubbles under the carapace of the exposed *D. magna*. Later on, another study compared the toxicity of multidimensional AgNMs (Ag nanospheres, AgPLs, and AgNWs) in *D. magna* and *Dapnhia galeata* (Cui et al., 2017). The authors confirmed the toxicity in the following order: AgPLs > Ag nanospheres > AgNWs. Although the same pattern was observed in both species, *D. galeata* resulted in more sensitivity to all tested NMs compared to *D. magna*, evidencing that the sensitivity of each species must be considered.

Another important physicochemical property of AgNPs that affects their toxicity includes surface coating. Poynton et al. (2012) found that Cit-AgNPs were more toxic to adult *D. magna* (LC_50_-24 h) than the PVP-AgNPs (Table 1), even though the size of the coated particles was similar, and they exhibited comparable dissolution rates of approximately 10%. However, the hydrodynamic diameter of the PVP-AgNPs was over twice the size of the Cit-AgNPs; therefore, the higher toxicity of the Cit-AgNPs may be related to the greater surface area of those particles. Similar results were obtained by Hou et al*.*, who correlated LC_50_ data with molecular mechanisms of differentially expressed genes. According to the authors, the lower toxicity exerted by PVP coated might be due to its good stability, which was confirmed by zeta potential values, a lower particle dissolution rate, and no obvious changes in cluster or aggregate formation.

Some studies have shown that the presence of dissolved organic matter (DOM) with humic and fulvic acids affects the stability and dissolution of AgNPs, with the consequent impact on its bioavailability and toxicity. For instance, the toxicity of AgNPs to *Ceriodaphnia dubia* and *D. magna* decreased in the presence of river humic acid or lake fulvic acid, respectively (Gao et al., 2012; Jung et al., 2018). Most of the toxicological studies mentioned above have been carried out using standard exposure media with limiting reliability of real exposure scenarios and consequent AgNP environmental transformations. However, some recent studies have addressed this issue by using natural exposure media. For example, Conine et al. (2017) assessed the toxicity of AgNPs to *Daphnia dentifera* in natural waters from six lakes. The authors found that LC_50_ values were extremely variable among all lakes (Table 1). Seasonality (in terms of animal life cycles) and variability in water and particulate chemistry (carbon:nitrogen ratio) were the primary variables with the greatest influence on survival rates. To a lesser degree, other variables in both the dissolved and particulate forms were also able to alter toxicity, including DOC, total dissolved phosphorus, and particulate carbon:phosphorus ratios. These results demonstrate the importance of understanding how multiple variables function together at an ecosystem scale.

On the other hand, some studies compared the impact of pristine medium (American Society for Testing and Materials, ASTM) and wastewater-borne exposed AgNPs on *Daphnia* and found that environmentally relevant concentrations of wastewater-borne AgNPs provoked a reduction of toxicity to *D. magna* (Hartmann et al., 2019; Galhano et al., 2020). This could be explained by chemical transformations of AgNPs (sulfidation and/or complexation with other ligands) during the lab-scale WWTP processing.

#### Bivalves and Snails

Molluscs play a crucial role in ecosystem functioning (e.g., nutrient and energy cycling through the food web), and they have several characteristics that make them valuable test organisms (ubiquity, sedentary life habit, availability, etc.) (Justice and Bernot, 2014). However, studies evaluating the effects of ENM/Ps in freshwater bivalves and snails are scarce compared to other representing groups such as fish, algae, and microcrustaceans (Gonçalves et al., 2017) (Figure 3).

The ecotoxicological risks for a wide range of ENM/Ps to bivalve and snails have been reviewed by Rocha et al. (2015) and Caixeta et al. (2020), respectively. Both reviews evidence disparity among studies using marine and freshwater species. Rocha et al. (2015) showed that evaluation of toxic effects of ENM/Ps in bivalves was conducted mainly with seawater (85%) species compared to freshwater ones (15%). On the contrary, most of all studies involving ENM/Ps and snails were performed with freshwater species (74%) when compared with land (18%), estuarine (6.5%), and marine (1.5%) species (Caixeta et al., 2020). Despite the increasing number of reports on NM ecotoxicity to freshwater molluscs, incomplete information is currently available regarding the toxicity of AgNPs.

As previously discussed, after agglomeration and sedimentation, AgNPs may ultimately accumulate in sediments. Jiang et al. (2017) demonstrated that the surface layer of sediment on an aquatic system in microcosms was the main sink of Ag originating from both AgNPs and AgNO_3_. This deposition of AgNPs on sediments may present a risk of exposure to sediment-dwelling organisms. So, benthic species are expected to be the main receptors of NPs (Klaine et al., 2008). Liu et al. (2018) determined the Ag concentration in water, sediment, and the bivalve *Corbicula fluminea* after a pulse treatment of AgNPs (added into upper water). The majority of Ag was deposited in sediment and then differently bioaccumulated in bivalve tissues: Ag tended to bioaccumulate in soft tissues at lower AgNP concentrations; on the contrary, Ag was more adsorbed on shells at higher exposure concentrations. This difference may be explained by a self-protection mechanism of *C. fluminea*, which closed shells. In addition, non-Ag was detected in faeces, probably indicating that AgNPs had a long gut retention time in this bivalve species. Similarly, *Lymnaea stagnalis* and *Biomphalaria glabrata* snails efficiently accumulated Ag from ionic and nanosized forms after either aqueous or dietary exposure, but the metal elimination was very slow (Croteau et al., 2011; Oliveira-Filho et al., 2019).

On the other hand, only few studies have assessed the toxic effect of AgNPs on freshwater molluscs using the sediment matrix (Bernot and Brandenburg, 2013; Bao et al., 2018; Liu et al., 2018). For instance, in the snail *Bellamya aeruginosa* exposed to AgNPs and AgNO_3_-spiked sediment, Ag burden varied with Ag form (aqueous > particulate) and tissue (hepatopancreas > gonad ≈, digestive tracts > foot) (Bao et al., 2018). The highest bioaccumulation factor (BAF) was obtained in the hepatopancreas, confirming it as the major tissue for Ag accumulation.

Interestedly, Bernot and Brandenburg (2013) determined the acute lethal toxicity (LC_50_) of AgNPs to *Physa acuta* in the absence and presence of sediment. The survival was higher when experimental flasks included sediment than in its absence (Table 1), suggesting that sediment in freshwater media may reduce the concentration of AgNPs or Ag ions that snails may encounter. On the other hand, Luoma et al., (2016) compared uptake rates of Ag in the *L. stagnalis* snails after AgNP exposure in the presence or absence of either humic acid (HA) or thiol-rich organic matter (e.g., cysteine). Ag uptake into snail tissues was not highly affected by the presence of HA. On the contrary, the Ag uptake from AgNP markedly decreased when cysteine was added to the media.

Besides NOM, AgNP stability and bioavailability are highly dependent on other environmental factors (i.e., electrolytes, hardness, and pH), which vary significantly among different aquatic systems (stream, lagoon, and river). In this way, Gonçalves et al. (2017) compared the acute toxicity of AgNPs and AgNO_3_ in adult and juvenile snails (*Physa acuta*) exposed to media with different chloride concentrations (equivalent to an environmental low and medium level). The results showed that juveniles were more sensitive than adults and that lethality was higher in the lower chloride level for both ionic and NP forms. Probably, the complexation between chloride and silver ions lead to reducing Ag^+^ bioavailability, uptake, and toxicity. Moreover, the higher-chloride-level medium led to a higher AgNP aggregation/agglomeration state, which would reduce their bioavailability.

Another aspect of water quality influencing the bioavailability and toxicity of AgNPs is the water hardness. Water hardness influenced the uptake rates of Ag from AgNPs in the freshwater snail *L. stagnalis* after short waterborne exposures (Stoiber et al., 2015). Namely, harder water increased aggregation, reducing the bioavailability of Ag. In a similar experiment, *L. stagnalis* efficiently assimilated Ag from AgNPs mixed with diatoms (dietary exposure), but water chemistry (hardness and humic acids) had little or no impact on AgNP bioaccumulation and toxicity (Oliver et al., 2014).

As already mentioned above, particle shape might influence AgNP toxicity. Recently, Auclair et al. (2021) investigated if different forms of AgNPs (sphere, cube, and prism) could initiate biophysical chenges (levels of liquid crystals (LCs) and changes in the activity and fractal dimensions of pyruvate kinase–lactate dehydrogenase (PK–LDH), F-actin, and protein ubiquitin (UB) levels) in mussels (*Dreissena bugensis*). The results suggested that the geometry of AgNPs could influence the formation of LCs (prismatic > cubic > spherical), alter the fractal kinetics of the PK–LDH system (spherical > cubic > prismatic) and F-actin levels, and cause protein damage (pattern similar to LCs levels) in the soft tissues of freshwater mussels. Thus, the AgNP form has effects on the spatial (fractal) organization in the cytoplasm where biochemical reactions take place.

In general, AgNPs effects are often size dependent, showing that smaller ones are more bioavailable and induce higher toxicity. However, this rule does not seem applicable to freshwater bivalves and gastropods. Bao et al. (2018) observed that larger AgNPs (40 and 80 nm) induced higher oxidative stress to snails compared with the smaller ones (20 nm). The authors hypothesized that 20 nm AgNPs were prone to enter or adsorb into the sediment pores (average pore size of sediment of 14.6 ± 7.7 nm). Therefore, the smaller particles cannot easily contact the snail and induce further toxicity. On the other hand, Gagné et al. (2013) examined the bioavailability and effects of AgNPs of two different sizes (20 and 80 nm) and Ag^+^ on the immune system, oxidative stress, and metal metabolism (metallothioneins and labile zinc) of the mussel *Elliptio complanata*. Results showed that Ag^+^ was more bioaccumulated than 20 or 80 nm AgNPs, but the global adverse responses of 20 and 80 nm AgNPs were similar to those found for Ag^+^ and independent of Ag particle size.

According to the literature review, the main AgNP biological effects in freshwater molluscs are oxidative stress, genotoxicity, reproductive and embryotoxicity, and behavioural impairment. AgNPs induced oxidative stress and DNA-damaging effects in the digestive gland (Ali et al., 2014) and hemolymph (Ali, 2014) of *L. luteola* snail. In addition, AgNPs reduced hemocyte viability and increased apoptosis and necrosis in a time-dependent concentration, suggesting that genotoxicity is mediated by oxidative stress (Ali, 2014). Thus, the authors suggested that oxidative stress may be involved in DNA damage induction, leading to apoptosis or causing cell death. Moreover, AgNPs induced oxidative stress in tissues of the mussels *E. complanata* and *C. fluminea* (Gagné et al., 2013; Liu et al., 2018).

Chronic exposure to AgNPs affects the reproduction rate and the early developmental stages of mollusc species. For instance, low concentrations of AgNPs (<1 μg L^−1^) resulted in decline in size at the first reproduction and egg production of *P. acuta* (Bernot and Brandenburg, 2013). A significant reduction in the number of eggs and embryo per individual was also observed in *G. glabrata* and *Potamopyrgus antipodarum* exposed chronically to AgNPs (Völker et al., 2013; Oliveira-Filho et al., 2019). Some authors compared the toxicity of AgNPs and Ag ions to *P. acuta* and *B. glabrata* (Gonçalves et al., 2017; Araújo et al., 2020). The results showed that both Ag forms increased the mortality, reduced the hatching, and augmented the frequency of hydropic malformation. However, AgNPs cause lower reproductive and embryotoxicity than their ion counterparts. This dissimilar toxicity could be explained by the mucous or gelatinous surface of eggs and embryos, preventing a massive penetration of the AgNPs.

On the other hand, non-lethal concentrations of AgNPs may alter mollusc behaviours, affecting food location, predator avoidance, searching mates, and habitat use, among other consequences. *P. acuta* habitat use was modified by AgNP exposure, with a higher proportion of gastropods occupying the near-surface habitat, which indicates avoidance behaviour (Justice and Bernot, 2014). In addition, AgNPs impaired *P. acuta* ability to respond to the presence of a fish predator cue by interfering with chemoreception (Justice and Bernot, 2014). According to Young et al. (2017), snails detect the presence of AgNPs with peripheral sensory receptors, and possibly, the NP acts as an irritant on sensory structures, which causes a stress response that alters the snail’s ability to form memory. Specifically, AgNPs affected cognitive processes in *L. stagnalis*, modifying its ability to learn and remember. However, results depended on the assayed concentration, where memory formation was blocked (50 μg L^−1^), enhanced (10 μg L^−1^), or not altered (5 μg L^−1^).

In summary, as shown for other freshwater taxa, the uptake, bioaccumulation, and biological effects of AgNPs in molluscs might depend not only on the intrinsic characteristics of AgNPs (e.g., shape and size) and on the environmental factors determining their fate and transformation (water quality) but also on the exposure routes (dietary, waterborne, and sediment) and other experimental conditions (time, concentrations, etc.).

#### Fish

Fish are widely used as test organisms for nanotoxicity research. According to a previous bibliometric analysis, the studies about the toxic effects of ENM/Ps in fish were conducted mainly with freshwater species compared with marine ones and *Danio rerio* (zebrafish) as the most common fish species used as a model system (∼60% of the total reviewed scientific articles) for testing ENM/P toxicity (Cazenave et al., 2019). Some reviews have even focused on the toxic effects induced by ENM/Ps in zebrafish and summarized the advantages of using this species for nano(eco)toxicity assessments (Chakraborty et al., 2016; Haque and Ward, 2018; Jia et al., 2019; Pereira et al., 2019; Bai and Tang, 2020).

When AgNPs come into contact with fish, they might be retained in the skin/gill mucus layer or be absorbed by either gills or intestinal epithelia and distributed into different tissues. The uptake and accumulation of Ag ions in the gills, liver, intestine, and brain have been reported by several studies (Wu and Zhou, 2013; Jung et al., 2014; Bacchetta et al., 2017; Ale et al., 2018a, Ale et al., 2018b; Khosravi-Katuli et al., 2018, among others). In the same way, it has been proved that AgNPs were able to penetrate zebrafish embryos through the chorionic pore (Lee et al., 2012) and that they were distributed in the brain, heart, yolk, and blood of embryos (Asharani et al., 2008).

There is a lot of evidence showing that AgNP exposure generates negative impacts on fish, although it is still discussed if the toxicity is mainly attributed to the release of silver ions (Ag^+^) or AgNP themselves (McShan et al., 2014; Wang et al., 2015; Cáceres-Vélez et al., 2018; Haque and Ward, 2018; Bai and Tang, 2020). Anyway, it has been shown that AgNPs can cause acute toxicity (Griffitt et al., 2008) and, mainly, a wide range of sublethal damages in fish. Interestingly, AgNPs provoked changes in the beneficial microbial community living on both skin and intestinal mucus, which constitutes the first barrier and immunological defence of fish (Merrifield et al., 2013; Bacchetta et al., 2016; Ale et al., 2018a). To counteract the effects of AgNPs, an increase in the number of mucus cells in the gills of *Cyprinus carpio* and *Prochilodus lineatus* has been recorded (Lee et al., 2012; Ale et al., 2018a).

Once AgNPs have been internalized by the cells, they elicit a range of toxicities, including immunotoxicity, cytotoxicity, genotoxicity, and other physiological effects. Undoubtedly, oxidative stress is the most studied mechanism for explaining AgNP toxicity. Exposure to AgNPs commonly results in the overproduction of ROS, the activation of the antioxidant system, the increase of lipid peroxidation, and membrane damage, which lead to apoptosis and loss of cellular functions (Chae et al., 2009; Lee et al., 2012; Massarsky et al., 2014; Valerio-García et al., 2017; Ale et al., 2018a; Ale et al., 2018b; Khan et al., 2018). On the other hand, some studies have demonstrated that AgNPs can reach the cell nucleus, inducing DNA damage and nuclear abnormalities (Sayed, 2016; Bacchetta et al., 2017; Sayed and Soliman, 2017).

In addition to cellular, subcellular, and molecular effects, the toxicity of AgNPs has been demonstrated at multiple levels on different toxic endpoints, such as diverse physiological processes (e.g., metabolic pathways, ion regulation, and respiratory system) (Bilberg et al., 2012; Massarsky et al., 2014; Valerio-García et al., 2017; Ale et al., 2018b; Khan et al., 2018), organ histopathology (Govindasamy and Rahuman, 2012; Lee et al., 2012; Rajkumar et al., 2016; Ale et al., 2018a; Khan et al., 2018), neurotoxicity and behavioural alterations (Powers et al., 2011), and embryotoxicity and teratogenic effects (Yoo et al., 2016; Pereira et al., 2019).

As already described for other aquatic organisms, smaller AgNPs seem more toxic, probably because of being more easily taken up by embryos or fish tissues. For instance, 4 nm AgNPs were more efficiently accumulated and resulted in higher zebrafish embryotoxicity than 10 nm particles (Xin et al., 2015). This is in line with the reported values of LC_50_ for adult zebrafish (Table 1), which clearly showed that smaller particles (20 nm) were more toxic than larger ones (100 nm), irrespective of the surface coating (Cit or PVP) (Liu et al., 2019). In another study, juvenile female rainbow trout (*Oncorhynchus mykiss*) exposed *via* water to nanosized (10 and 35 nm) and bulk-sized silver particles (600–1,600 nm) for 10 days showed that the smaller particle was the most highly concentrated in the gills and induced expression of genes involved in oxidative metabolism (Scown et al., 2010). However, the liver analysis did not show differences in Ag concentration between 10 nm AgNP and bulk silver treatments. Similarly, Osborne et al. (2015) investigated the size-dependent effects between 20 and 110 nm of AgNPs in the zebrafish gills and intestine, utilizing different endpoints. Results showed that 20 nm AgNPs were more accumulated in gills than the 110 nm ones, while the Ag content was similar in the intestines for both particle sizes. Moreover, AgNPs were localised in the basolateral membranes, leading to disruption of the Na^+^/K^+^ ion channel in both target organs, with the smaller particle causing greater toxicity than the larger one. At the embryonic stage, mortality, morphological malformations, and altered locomotor activity were also observed after different sizes of AgNPs’ exposure (Bar-Ilan et al., 2009; Powers et al., 2011; Osborne et al., 2013). However, toxicity was size dependent at certain concentrations, times, and light conditions. Thus, the size dependence of AgNP-mediated toxicity is still controversial (Haque and Ward, 2018).

On the other hand, some researchers have also studied the relationship between AgNP shape and toxicity. George et al. (2012) compared nanosized Ag spheres, plates (AgPLs), and wires (AgNWs) in both *in vitro* (rainbow trout gill fish cell, RT-W1) and *in vivo* (zebrafish embryos) models. Results showed that AgPL induced higher cyto- and embryotoxicity than other particle shapes in spite of a lower rate of dissolution and bioavailability of plate-shaped Ag. The authors demonstrated that these differences were due to a high level of crystal defects on the AgPL surfaces, which caused damages during direct physical contact with the cells or organism. Thus, in addition to Ag ion release, the increased surface reactivity of AgPL should be considered as another important mechanism of nanosilver toxicity (George et al., 2012). In agreement with this study, Abramenko et al*.* (2018) reported higher acute toxicity of AgPL to *D. rerio* embryos than nanospheres (Table 1). Interestingly, both types of NPs were more toxic than silver ions (LC_50_ 0.0649 mg L^−1^), suggesting that toxicity may be associated with AgNPs themselves rather than with ionic silver released into solution. In addition, the LC_50_ values of Ag nanospheres and AgNW for *Oryzias latipes* indicated that nanospheres displayed greater toxicity than NWs (Table 1).

In agreement with other freshwater taxa, surface modifications of AgNPs could also influence the toxicity of AgNPs in fish. For instance, Osborne et al. (2013) demonstrated that coating AgNPs with Cit or fulvic acid reduced mortality rates in zebrafish embryos compared to uncoated NPs. In another study, AgNPs coated with gum arabic exhibited more toxicity than PVP and Cit AgNPs (which showed similar and lower toxicity, respectively) to early life stages of *O. latipes* (Kwok et al., 2012). However, these results should be interpreted with caution because differently coated AgNPs showed dissimilar aggregation behaviour and dissolution in the test medium. On the other hand, Auclair et al. (2019) compared the bioavailability and toxicity of AgNPs having similar size and shape (50 nm, spherical), but different coatings: Cit, PVP, branched polyethyleneimine (bPEI), and silicate (Si). Although Ag was detected in the liver of *Oncorhynchus mykiss* juveniles from the four treatments, some coatings led to more bioavailability than others (PVP > Cit > bPEI > Si); for example, the hepatic Ag content of AgNP-PVP was 15 times higher than that of AgNP-Si. Regarding toxicity, the authors showed that both negative (Cit) and positive (bPEI) coatings caused more DNA damage and inflammatory effects than neutral coatings (Si and PVP). Thus, these results demonstrated that NP charge, which is conferred by the coating, might be an important intrinsic particle property governing toxicity. In line with these results, Liu et al. (2019) reported higher Ag contents in different organs (intestines > gills > muscles) of zebrafish after exposure to AgNP-PVP compared to AgNP-Cit. In contrast, acute toxicity tests and gene expression analyses confirmed that AgNP-Cit were more toxic. Differences in toxicity could be caused by changes in zeta potential. In this regard, the authors observed that the absolute zeta potential on the surfaces of Cit decreased after 96 h, while only slight changes were observed for PVP, indicating that AgNP stability played an important role in affecting biological responses.

Interestingly, Lee et al. (2013) synthesized AgNPs, which were functionalized with peptides in order to offer positive (3.0 ± 0.2 mV) or negative (−5 and −11.9 mV) charges. The three Ag-peptide-NPs were very stable in zebrafish exposure media over the entire duration of the experiment (120 h), and they passively diffused into embryos *via* chorionic pores and affected embryonic development. However, positively charged AgNPs diffused faster and longer distances than negatively charged ones, suggesting that the first ones were more biocompatible. In contrast, the most negatively charged NPs (−11.9 mV) caused the highest mortality rate (77%), while positively charged AgNPs showed the lowest toxic effects (0% deformed embryos; 33% mortality). Thus, the biocompatibility and toxicity of AgNPs could be dependent on their surface charges.

Concerning the development of the environmentally friendly synthesis of ENM/Ps, it is demonstrated that AgNPs produced from the green methods are usually less toxic than those achieved from the non-green methods. Moreover, according to the literature reviewed by Tortella et al. (2020), AgNPs biosynthesized from plant extracts (e.g., *Ocimum tenuiflorum* and *Brassica oleraceae*) (Daniel et al., 2011; Lathamuthiah et al., 2015) seemed to be less toxic to zebrafish eggs than those synthesized by *Escherichia coli* (Kannan et al., 2011). However, further studies are necessary due to other factors, such as the size or the presence of capped protein, which might also explain the differences in toxicity.

In addition to the different properties of the particles, several experimental or environmental conditions could influence AgNP toxicity. For example, George et al. (2014) observed that simulated solar light induced transformation of AgNPs (surface oxidation and Ag^+^ release) and augmented their toxicity to zebrafish embryos. On the other hand, exposure medium containing a high concentration of chloride ions (Groh et al., 2015) or higher salinity (Kalbassi et al., 2011; Cáceres-Vélez et al., 2018; Joo et al., 2018) attenuated the acute and sublethal effects of AgNPs to zebrafish embryos and rainbow trout fry. Taken together, these findings suggest that laboratory conditions may over- or under-estimate the risk potential of ENM/Ps and demonstrate the need for carrying out toxicity tests under environmentally relevant exposure conditions.

Similarly, it was demonstrated that the presence of natural organic matter (NOM) affects the physicochemical properties of AgNPs (e.g., initial diameter, surface charge, and dissolution rate), leading to a lower bioavailability and toxicity. For instance, the EC_50_ values, based on morphology and teratogenicity endpoints in zebrafish embryos, showed that the toxicity of AgNPs decreased with increasing HA substances concentrations (Wang et al., 2015). In the same way, Cáceres-Vélez et al., 2018; Cáceres-Vélez et al., 2019 reported that the presence of HA in an exposure medium (zebrafish facility water) reduced the mortality (Table 1), adverse effects, and Ag content in the embryos and adults of zebrafish exposed to AgNPs (Cáceres-Vélez et al., 2018; Cáceres-Vélez et al., 2019). In addition, Kim et al. (2013) demonstrated that the interaction of AgNPs with HA mitigated the mortality of *Oryzias latipes* embryos. On the other hand, Ale et al. (2021a) and Ale et al. (2021b) assessed the mitigation of HA on bioaccumulation and oxidative stress in the gills of Neotropical fish (*Piaractus mesopotamicus* and *Corydoras paleatus*) exposed *ex vivo* to AgNPs. *P. mesopotamicus* gills accumulated Ag almost six-fold lower when HA was present in the media. Moreover, AgNP treatment (without the presence of HA) increased catalase activity in the *P. mesopotamicus* gills and the lipid peroxidation levels in *C. paleatus*. However, these oxidative stress responses were similar to control values when the HA was added to the media. In addition, the authors observed that HA was adsorbed on the AgNPs surface and reduced the Ag^+^ release, impacting the nanoparticle fate and toxicity.

### Brackish and Marine Environments

The ecotoxicity of AgNPs and Ag-based NEPs for marine species has been less investigated, and MoA, barely understood if compared to freshwater ones (Butz et al., 2019), was also confirmed by the wide range of reported effect concentrations (EC_50_ from 1 μg L^−1^ to >100 mg L^−1^, see Table 1) which often barely mimic real exposure scenarios.

#### Phytoplankton

Several studies have been reporting the ability of AgNPs to inhibit cell growth in marine phytoplankton including diatoms (Burchardt et al., 2012; Angel et al., 2013; Gambardella et al., 2015; Huang et al., 2016; Sendra et al., 2017; Pham, 2019), green microalgae (Oukarroum et al., 2012; Gambardella et al., 2015; Sendra et al., 2017; Hazeem et al., 2019), raphidophytes (He et al., 2012), and cyanobacteria (Burchardt et al., 2012) with effect concentrations in the range from μg L^−1^ to mg L^−1^ (see Table 1 for details) depending on the specific size, coating, and surface charges of AgNPs stock tested and the taxa investigated.

In a recent study by Dedman et al. (2020), microbial cell density seemed to affect the toxicity (populations reduced by over 90%) and recovery of the cyanobacteria *Prochlorococcus* strain MED4 exposed to Cit-stabilized AgNPs (≥10 μg L^−1^) under environmentally relevant cell densities in natural oligotrophic seawater. Synergistic adverse effects caused by AgNPs (e.g, induced oxidative stress) as a consequence of NP–cell membrane interaction and dissolved Ag ions are hypothesized. Oxidative stress and damage on cell walls or membrane is largely documented in phytoplankton upon AgNP exposure due to the production of ROS and/or release of Ag ions. Photosynthesis is also reported to be affected generally as a decrease in chlorophyll-α content and lipid peroxidation in the marine diatom *Talassiosira* sp. (0–200 μgL^−1^) (Pham, 2019) and in the microalgae *Chlorella vulgaris* (100–200 mgL^−1^) (Hazeem et al., 2019), *Dunaliella tertiolecta* (10 mgL^−1^) (Oukarroum et al., 2012), and *Phaeodactylum tricornutum* (10–300 μg L^−1^) (Sendra et al., 2017) and disruption in photosystem-II electron transport in the marine diatom *Skeletonema costatum* (AgNPs coated with oleylamine, 0.5 ml^−1^) (Table 1) (Huang et al., 2016).

Cell wall composition/structure and coating agents have been recognized as the main drivers of algal toxicity in three marine microalgae (*Isochrysis galbana, Phaeodactylum tricornutum*, and *Tetraselmis suecica)* exposed to PVP/bPEI-coated AgNPs (5 nm) and uncoated (47 nm). Schiavo et al. (2017) suggested that the coating agent was most responsible for the observed algal toxicities as well as their different sensitivities based on the presence of a resistant silicified cell wall in the diatom.

Coating agents can control particle dissolution and, thus, the release of Ag ion, for instance, by limiting the particle contact with oxidizing agents, such as dissolved oxygen and ROS or driving aggregation (Gondikas et al., 2012; Sigg and Lindauer, 2015; Wu et al., 2017). Examples of reduced particle dissolution and ecotoxicity have been provided with AgNPs coated with sulfur and sulfur-containing molecules (Yang et al., 2012; Levard et al., 2013) such as cysteine (Prosposito et al., 2019).

As already mentioned for freshwaters, an increase in ionic strength as the one of seawater has been shown to play a significant role in increasing AgNP dissolution though this is not associated with an increase in ecotoxicity due to complexation of Ag ions with chloride species and the presence of NOM rich in sulfur and nitrogen (Kennedy et al., 2012; Gunsolus et al., 2015; Asadi Dokht Lish et al., 2019; Li et al., 2020). Therefore, in seawater, AgNP ecotoxicity for single-cell species seems to be the result of more complex dynamics, somehow higher than expected based on only Ag ions’ dissolution rate and supporting the theory of a NP-specific effect.

#### Microcrustaceans

Falugi et al. (2012) first reported AgNP (1–10 nm) toxicity in the range between 1.0 × 10^–1^ mg L^−1^ and 100 mg L^−1^ for brine shrimp *Artemia salina* using serial dilutions. Higher toxicity for the brine shrimp was reported upon exposure to PVA-stabilized AgNPs (EC_50_ 5.5 × 10^–2^ mgL^−1^) (Becaro et al., 2015).

The acute exposure of *A. salina* nauplii (72 h) to AgNPs (<100 nm; 0.39, 1.56, 6.25, 25, and 100 mgL^−1^) and AgNWs (0.01, 0.1, 1, 10, and 50 to 100 mgL^−1^) caused concentration-dependent immobilization with a clear sign of Ag retention inside the gut and adsorption in the external whole body of the nauplii (An et al., 2019).

A concentration-dependent decrease in cyst hatching, immobilization, apoptotic cell and DNA damage, and the presence of aggregates in gut regions were observed in *Artemia* sp*.* nauplii upon exposure to AgNPs (30–40 nm) (Arulvasu et al., 2014). Gambardella et al. (2015) reported concentration-dependent sensitivity and effect upon exposure to AgNPs (1–10 nm, 1–1,000 μL^−1^) in marine species belonging to different trophic levels as microalgae *D. tertiolecta* and diatom *Skeletonema costatum*, the jellyfish *Aurelia aurita*, microcrustaceans *Amphibalanus amphitrite* and *A. salina*, and echinoderma *P. lividus* (Table 1). Brine shrimp were less sensitive compared to the other species which showed an increased sensitivity from diatoms, green algae, sea urchins, barnacles, and finally, jellyfish as the most sensitive.

A harmonized protocol for testing ENM/P ecotoxicity including AgNPs using the brine shrimp *Artemia franciscana* has been proposed by Kos et al. (2016). The intercomparison of laboratory data revealed poor repeatability of AgNP toxicity results probably caused by the variation in the illumination regime which could cause different Ag species to be formed, so a standardized 16 h/8 h light/dark cycle should be adopted. Moreover, incubation conditions of plates during the test (room T, incubator, and ventilation) could play a role in the ecotoxicity outcomes; therefore, the authors concluded that more studies should be performed to standardize such procedures. Similarly, Asadi Dokht Lish et al. (2019) reported how changes in environmental parameters affected AgNP (8.80 ± 5.13 nm, max 29.1 nm) effect concentration (EC) values for *A. salina* instar I, for instance, decreased with increasing water temperature, decreasing water salinity, and in continuous darkness condition; in addition, EC_50_ value decreased in specimens exposed in 100 ml beakers (21.35 ± 5.67 mgL^−1^) compared to 10 ml well plates (42.44 ± 11.30 mgL^−1^). A concentration-dependent manner of the immobilization rate of *A. salina* nauplii with higher sensitivity of instar stage II than instar I at higher concentrations (*p* < 0.05) was also reported.

#### Bivalves

Several contributions have investigated the ecotoxicity of AgNPs in marine benthic filter-feeders as bivalves, as they are recognized among the most suitable marine bioindicators of anthropogenic pollutants including the emerging ENM/Ps (Matranga and Corsi, 2012; Canesi and Corsi, 2016). *In vivo* studies mostly concentrated on the understanding of potential bioaccumulation through waterborne exposure and biological effects through physiological and molecular biomarkers in potential target tissues (e.g., gills, immune circulating cells as hemocytes, and digestive glands) (Ringwood et al., 2010; Buffet et al., 2013; Gomes et al., 2013a, Gomes et al., 2013b; McCarthy et al., 2013, Gomes et al., 2014; Buffet et al., 2014; Bebianno et al., 2015; Katsumiti et al., 2015; Jimeno-Romero et al., 2017). Omics studies have also been performed to unravel the mechanism of actions and pathways of exposure by transcriptomics and proteomics coupled with physiological responses. In a more recent study by Duroudier et al. (2019a), mussels (*Mytilus galloprovincialis*) exposed to 10 µgL^−1^ 5 nm PVP/bPEI-coated AgNPs through diet (microalgae) in autumn and spring showed Ag bioaccumulation after 21 days in the digestive gland and gills and proteomics recognized 104 differentially expressed protein spots in autumn and 142 in spring suggesting how season could affect organism responses to AgNP exposure. The same authors showed that, upon 21-day dietary exposure (by microalgae) to PVP/bPEI-coated 5 nm AgNPs (1 μg L^−1^–10 μg L^−1^), impairment of reproduction was observed with females releasing less eggs than non-exposed ones and higher % of abnormal embryos at both exposure concentration doses. Cytotoxicity on mussel’s hemocytes exposed *in vitro* for 24 h was also observed (Duroudier et al., 2019b). Changes in mussel’s gill and digestive gland proteome, but not in gene transcription profile, were previously reported upon exposure to either AgNPs (10 µgL^−1^ AgNPs for 15 days**)** or AgNO3, but with different expression signatures, thus suggesting the involvement of different mechanisms in the observed AgNP and Ag ion toxicity (Gomes et al., 2013a, Gomes et al., 2013b; Bebianno et al., 2015). In a similar study, the same authors showed Ag accumulation in both tissues (gills and digestive glands) and induction of antioxidant enzymes and metollotionein; however, in the digestive glands, only a small fraction of Ag seems to be associated with this protein as well as for lipid peroxidation more marked in the gills than in the digestive glands (Gomes et al., 2014). Bouallegui et al. (2017a) reported morphological changes associated to the inflammatory response in the gills of mussels exposed for a short time to PVP-coated AgNPs (3, 6, and 12 h) and the highest histopathological indices upon exposure to AgNPs of the smallest size (<50 nm). They concluded that inflammation intensity was related to NP size and exposure time and overall toxicity depending on the uptake pathway.

By testing a AgNP-based commercial product (nanArgen^™^,Nanotek S.A; Ag purity>99%, 20–40 nm, with PVP as the stabilizing agent), we showed strong similarities in marine mussel adverse effects with those reported for pristine AgNPs at even more realistic exposure concentrations (1 and 10 µgL^−1^, 96 h) (Ale et al., 2019). Again, mussel’s gills seemed the more affected by the disruption of ATP-binding cassette proteins’ functionality which regulate the efflux of other toxic chemicals from the cells, thus having a protective role during filtration and feeding. Ag ion bioaccumulation was also observed in agreement with previous studies with bare AgNPs (Gomes et al., 2013a; Gomes et al., 2013b). The effect of size (20, 40, and 100 nm) of maltose-stabilized AgNPs and commercial ones in comparison with ionic and bulk Ag was investigated by Katsumiti et al. (2015) on mussel immune cells (hemocytes) and gills showing higher cytotoxicity for the smaller size (20 nm) similar to ionic Ag probably as a consequence of higher dissolution of Ag. Similar responses upon maltose-stabilized AgNPs and Ag ion exposure were also observed in both cell types (hemocytes and gill cells) with induction of oxidative stress (e.g., ROS production and antioxidant enzymes catalase, CAT) and DNA damage, activation of lysosomal AcP activity, disruption of actin cytoskeleton, and stimulation of phagocytosis in hemocytes and increase of MultiXenobiotic Resistance (MXR) transport activity and inhibition of Na-K-ATPase in gill cells. DNA damage on mussel hemocytes (comet assay) was already reported by Gomes et al. (2013a) upon exposure for 15 days to AgNPs (<100 nm) and bulk form, the latter showing higher genotoxicity suggesting a different mechanism of action probably mediated by oxidative stress. Silver ion dissolution of 20 nm maltose-stabilized AgNPs (tested in the range 0.75, 75, and 750 μg L^−1^) was recognized responsible of edema/hyperplasia in the gills and severe damage on mussel digestive tissues upon 21 days of exposure associated with intralysosomal Ag accumulation, lysosomal membrane destabilization, loss of digestive gland integrity, vacuolization in digestive cells, and atrophy–necrosis in digestive alveoli (Jimeno-Romero et al., 2017). Upon chronic exposure to AgNPs obtained from a green synthesis method using *Ceratonia siliqua* fruit extract to the clam *Ruditapes decussatus in vivo* for 30 days (1, 2.5, and 5 mg/aquarium), an increase in glutathione transferase (GST), glutathione reductase (GR), and CAT activities was observed in the gills (Hidouri et al., 2017). More recent findings on the same species on short-term exposure (48 h and 7 days) to commercial AgNPs (20–30 nm, 100 and 200 μg L^−1^) confirmed the gills’ antioxidant system as a target of AgNPs with changes in CAT significantly varying depending on AgNP concentration, time of exposure, and organ analyzed. On the contrary, GST and acethylcholinesterase (AChE) activities significantly decreased, thus hypothesizing an antagonistic effect on clams in both oxidative and cholinergic states (Elyousfi et al., 2021).

Dissolution of Ag ions as the main driver of toxicity of AgNPs was also hypothesized for endobenthic marine bivalve *Scrobicularia plana* upon exposure to 10 µgL^−1^ AgNPs (40 nm) and bulk Ag for 14 days either directly (water) or *via* the diet (microalgae) (Buffet et al., 2013). However, based on results on DNA damage and phenoloxidase and lysozyme activities in digestive glands of *S. plana* caused by AgNPs and bulk forms upon 21 days of exposure in mesocosms, the same authors recognized a nano-specific Ag effect (Buffet et al., 2014). No differences in Ag levels were observed between mesocosms contaminated with AgNPs and soluble Ag as well as bioaccumulation in specimens exposed to AgNP aggregates and ionic forms. A different tissue response towards 20–30 nm Cit-coated AgNPs and bulk Ag forms is reported for *Crassostrea virginica* with oxidative damage in the gills upon exposure to dissolved Ag and in the digestive glands to AgNPs with consequences on metabolism and reproductive impairments, suggesting a distinct NP effect (McCarthy et al., 2013). More recently, Carrazco-Quevedo et al. (2019) reported higher dissolution rate of low concentrations of AgNPs NM300K (20 ± 5 nm) (12.5 µgL^−1^ vs 125 µgL^−1^) in the presence of the rock oyster *Saccostrea glomerata*, with higher bioaccumulation in the gills, but not the digestive gland and induction of DNA strand breaks and oxidative stress biomarkers (GST, GR, and lipid peroxidation (LPO)). The authors underline the importance of characterizing the bio-nano-interaction of AgNPs in seawater media which are affecting dissolution and aggregation, thus influencing the uptake and bioaccumulation of AgNPs, including aggregates.

#### Benthic-Grazers

Few contributions have been made on marine benthic-grazers such as sea urchins. Magesky et al. (2017) reported apoptotic-like processes in the immune cells of *Strongylocentrotus droebachiensis* upon exposure to poly(allylamine)-coated AgNPs (17 ± 6 nm, PAAm-AgNPs) at 100 µgL^−1^ up to 96 h. By comparison with the ionic form, a differential uptake is hypothesized as mediated by a *Trojan-horse* mechanism operating over a 12-day exposure to AgNPs, while physical interactions with cell structures in short-term are responsible for the highest levels of stress-related proteins observed. The same authors also reported lower effect on the developing embryos of *S. droebachiensis* compared to Ag ions probably due to the low dissolution of PAAm-AgNPs in seawater (Magesky et al., 2017). In a previous study, the authors reported lower effect on mid-to-late gastrula to PAAm-AgNPs at 50 and 100 µgL^−1^ compared to Ag ions (Magesky and Pelletier, 2015). In the Mediterranean sea urchins *Paracentrotus lividus*, short-term exposures to 5–35 nm Cit-stabilized AgNPs (∼0.3 mgL^−1^) caused dose-dependent developmental abnormalities in embryos and larvae although no explanation on MoA is provided (Šiller et al., 2013). A different species sensitivity of sea urchin embryos to AgNPs (50 nm) was also revealed upon short-term exposure in the range of 1–100 μg L^−1^, with *Arbacia lixula* resulting the most sensitive with more aberrant embryos or arrested development at the lowest concentrations tested (1–10 μgL^−1^ AgNPs) compared to *P. lividus* and *Sphaerechinus granularis* (Burić et al., 2015). Sperm motility of *P. lividus* resulted to be affected upon AgNP exposure (Gambardella et al., 2015) (Table 1).

#### Sediment-Dwelling Species

Several contributions have been published on sediment-dwelling species in terms of the bioaccumulation and biological effects of AgNPs. In the work of Cong et al. (2014), Ag accumulation and effects of nano (<100 nm)-, micro (2–3.5 µm)-, and ionic (AgNO_3_)-Ag were investigated on the polychaete, *Nereis diversicolor.* The highest genotoxicity of the nano-Ag form compared to the other two was observed but no differences in the Ag bioaccumulation probably due to ingestion of Ag-associated particles in the sediment during feeding, or through body surface contact during burrowing. Higher DNA damage and protein activities related to immune system disruption have been also reported in *H. diversicolor* upon exposure to lactate- and maltose-coated AgNPs (40–45 nm, 10 µgL^−1^) compared to Ag ions (Mouneyrac et al., 2014). In outdoor mesocosm settings, Buffet et al. (2014) reported bioaccumulation of Ag upon exposure to AgNPs (10 µgL^−1^) and ionic Ag in *H. diversicolor* for 21 days and induction of oxidative stress defenses, detoxification, apoptosis, genotoxicity, and immunomodulation with higher phenoloxidase and lysozyme activities suggesting a specific nano-effect. Similarly, Cozzari et al. (2015) reported changes in antioxidant markers (glutathione, superoxide dismutase (SOD), CAT, glutathione peroxidase, GST, and GR) without significant Ag accumulation upon exposure to either AgNPs of different sizes (63 ± 27 nm; 202 ± 56 mm) or ions form Ag (range 2.5, 5, and 10 μg g^−1^sediment (dw)) in specimens of *Nereis diversicolor* exposed to contaminated sediments up to 11 days. Concentration- and time-dependent differences in the accumulation of the three Ag forms were observed, and the effects caused by AgNPs on biomarker responses suggest that the mechanism of oxidative stress is distinct from that of dissolved Ag.

Effects of AgNPs on early life stages are also reported on polychaetes. García-Alonso et al. (2011) reported mortality and abnormal development rate increased with early life stages in specimens of *Platynereis dumerilii* exposed to Cit-AgNPs or HA-AgNPs coated AgNPs and dissolved Ag. Exposures to HA-AgNPs caused higher toxicity responses and uptake rate in adult worms, consistent with higher toxicity in early life stages by AgNPs than in juveniles or adults. Chan and Chiu (2015) reported effects on growth, development, and settlement in the polychaete *Hydroides elegans* larvae upon exposure to oleic acid or PVP-coated AgNPs (30–50 nm, 1–100 μL^−1^). Sediment contaminated with either mixed Cit-AgNPs (13 nm) or ion Ag (100 µgg^−1^ dry weight) caused bioaccumulation of Ag but no related toxicity and differential impact on two polychaete species *Capitella teleta* and *Capitella* sp*.* Impairment of growth was reported for *Capitella* sp*.*, while *C. teleta* was not affected by either Ag form (Ramskov et al., 2015). A 28-day bioaccumulation study showed a different pattern of speciation upon the AgNP coating on bioaccumulated Ag in the marine polychaete *Nereis virens* upon exposure to Cit-AgNPs and PVP-AgNPs, as well as Ag ionic form. Such findings suggest the influence of coating agents on Ag uptake, biotransformation, and/or excretion in marine polychates (Wang et al., 2014).

#### Fish

Few studies have been performed on assessing the impact of AgNPs on marine/brackish fish species. Kleiven et al. (2019) reported that Ag is taken up regardless of the route of exposure (waterborne and diet) in juvenile of Atlantic salmon (*Salmo salar*) exposed to radiolabeled Cit-AgNPs (110 mAg) and Ag ionic form for 48 h. Higher Ag concentrations in the gills are observed *via* waterborne exposure, while dietary exposure led to high concentrations in the gastrointestinal tract, with the liver as the main target organ. Ionic Ag forms were highly accumulated through water, while no differences were observed in specimens exposed through diet. The smallest citrate-AgNPs (4 nm) resulted in four orders of magnitude more accumulation from water than from feed; otherwise, their transfer from other contaminated prey could provide long-term exposure scenarios with unknown consequences on Ag bioaccumulation and fish health.

Upon exposure to 5 nm PVP-AgNPs (80 μg L^−1^ for 48 h), adult killifish, *Fundulus heteroclitus*, showed a significant depression of oxygen consumption (ṀO_2_min and ṀO_2_max) compared to control fish, but neither aerobic scope nor biochemical indicators of toxicity were affected as well as gill epithelium (Campbell et al., 2019). Furthermore, gill NKA activity, although sensitive to Ag ions, did not affect specimens exposed to PVP-AgNPs suggesting that dissolution, even occurring, is not making Ag bioavailable to fish gills.

## The (Eco)safe by Design Approach to Prevent Human and Environmental Health Effects

Several progresses have been made in the last years towards the definition of successful strategies aiming to assess the nano(eco)safety of ENM/Ps as demonstrated by a number of publications and projects addressing “nanosafety” either for humans and the environment (Corsi et al., 2014, Corsi et al., 2021; Hjorth et al., 2017; Joint Research Centre European Commission, 2017; Lin et al., 2018; Chen et al., 2020). At the European level, the most recent “Safe and Sustainable-by-Design” (SSbD) intended as “a systems approach by integrating safety, circularity, and functionality of advanced materials, products, and processes throughout their life cycle” has been proposed with the aim to reduce any potential hazard posed by ENMs from their synthesis, production, application, and final release into the environment following the fundamental and well-developed framework of environmental risk assessment adopted for legacy pollutants (Gottardo et al., 2021). Also, the OECD recently developed a Safe(r) Innovation Approach (SIA) still relying on the safe by design concept along with life cycle assessment and socioeconomic analysis (SEA) (regulatory Ppreparedness) to be considered as a priority issue for limiting/reducing risks to humans and the environment (Soeteman-Hernández et al., 2019; OECD, 2020b).

Significant steps towards providing guidelines for ENM/P ecotoxicological testing which cannot follow classical molecules due to their peculiar nature and properties have been made by the OECD with a guidance document published in 2020 for freshwater aquatic and sediment toxicological testing (n. 317) (OECD, 2020b). Several important issues as the need to fully characterize particle properties and behaviour prior to test and during testing with organisms inside, as well as the metrology used to prepare test dispersion and recommendations on how to conduct test and how to analyse data and report them to the scientific community, have been faced and guidelines are provided.

A set of required minimum criteria for ENM/P characterization such as, for instance, size, shape, composition, and purity are already mandatory in nano(eco)toxicological testing in order to achieve a better comparison of experimental data and environmental metrics in the design process together with functional performances and costs (e.g., Nanotechnology Road Map for 2030) (Haase and Klaessig, 2018; Plata and Janković, 2021). Aquatic nanoecotoxicology has moved forward in such direction by including as mandatory a full characterization of environmentally relevant exposure conditions (e.g., aggregation, agglomeration, and eco-bio-corona formation in water media) to provide more consistent and reliable informative data for exposure and hazard estimates (OECD, 2020a).

**FIGURE 4:**
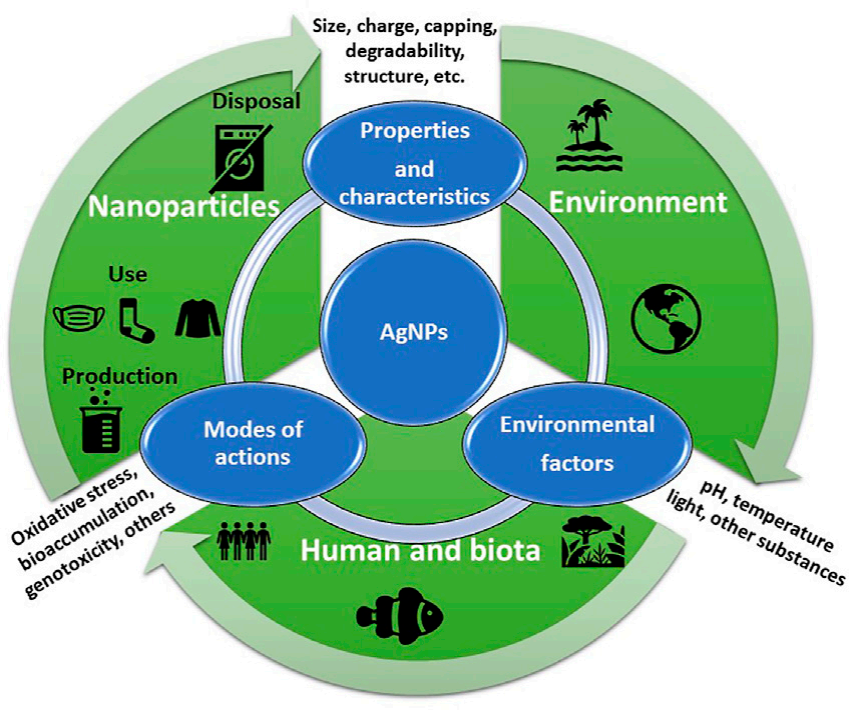
Schematic representation of the production, use, disposal, fate, and effect of AgNPs as well as the main parameters affecting the toxicity mechanisms of AgNPs.

In terms of environmental safety design named as *eco-design*, those ENM/P properties that will satisfy high safety standard for humans and environmental health should be incorporated into the material design since the beginning of their conceptualization while avoiding those which could represent a potential hazard from their synthesis (e.g., cradle) to their final disposal (e.g., grave) by following a re-design conceptual procedure (Fiorati et al., 2020a, Fiorati et al., 2020b; Corsi et al., 2021). The knowledge acquired by ecotoxicity testing of AgNPs in either freshwater or marine species allows us to recognize some peculiar properties of the NPs themselves which could be considered predictors of environmental safety such as, for instance, particle size, surface coating, and charges according to the specific properties of the water body receiving them (e.g, freshwater, brackish, or marine). The possibility to build up a validation framework in which the ENM/P properties along with ecotoxicological testing will be defined along with their efficacy towards specific application and final disposal into the environment could guide the product development before being put into the market, thus limiting any potential human and environmental risk (Figure 4).

As such, this safety approach can be recognized as a “precautionary strategy” which includes both human and environmental safety although not yet adopted but clearly supported as demonstrated by a number of funded projects by the European Commission in collaboration with international funding agencies and bodies, already showing promising results (www.nanosafetycluster.eu).

An example in this direction has been made in the development of sensors for mercury based on plasmonic AgNPs with the aim to support their safe environmental applications in both fresh and marine waters (Prosposito et al., 2019). The addition of specific capping molecules such as Cit and L-cysteine on the surface of AgNPs, thus increasing reduced sulfur groups (SH-) able to bind free cationic metals, was shown to successfully limit their ecotoxicity for either freshwater or marine microalgae. The sulfidation of AgNPs, with the formation of an insoluble Ag sulfide (Ag_2_S) layer on the surface of the NP, has been shown to significantly reduce its dissolution and increase aggregation (Levard et al., 2013). Lower ecotoxicity of NPs including AgNPs functionalized with sulphur groups has been reported compared to bare NPs (Levard et al., 2011, Levard et al., 2012; Pem et al., 2019). The release of silver ions in water media (either fresh and saline) and ecotoxicity assessed towards two model microalgae, such as the freshwater *R. subcapitata* and the marine *P. tricornutum,* demonstrated such design to be environmentally safe, ecosafe, in terms of reduced dissolution and ion release and no ecotoxicological effects.

The toxicological information from biological models represents a useful tool to predict the potential risk of nanomaterials on humans and the environment (Zielińska et al., 2020). However, due to the several different factors that influence the toxicity of AgNPs, more efforts from the scientific community, industrial companies, and governments are required. Physical and chemical synthesis procedures are widely employed, and their impact was a subject of investigation, but the emerging field of ecofriendly methods should be also taken into account to decrease the environmental impact of their production. In addition, the characteristics of the AgNPs could also be tuned to diminish their undesired effects. Initially, the size-dependent effect of NPs was well known and reported, suggesting that smaller AgNPs are more toxic probably due to their higher bioavailability and increased release of silver ions. Secondly, surface chemistry and charge were also identified as key parameters affecting the interaction with cell membranes, the adsorption of molecules, and the protein corona formation. In this sense, NPs with surface positive charge (zeta potential) were found to be safer. Finally, the capping agent employed is also highly relevant since it determines the stabilization of the AgNPs and also controls the release of silver ions. In conclusion, the criteria for the completely safe application of AgNPs are not easily met; however, great progress has been made toward this end. The generation of multidisciplinary forums of research with contribution of researchers of different fields will certainly contribute to developing the safety by design approach and, consequently, the successful application of AgNPs.

## Final Remarks

During the past few years, an interesting number of new applications of AgNPs have emerged. Indeed, the unique physical and chemical functions together with antimicrobial activities and new large-scale manufacturing procedures suggest that they have important applications. Moreover, considerable efforts have been devoted to analysing the sophisticated interactions of AgNPs with biological systems. The different studies presented in this review highlight the importance of these emerging fields with implications in human and biota healthcare. Finally, to minimize the human and environmental risks of ENM/P s, toxicity results need to be considered during their design and production. Building the bridge from aquatic nanotoxicology to *safety by design* of ENM/P made of silver may contribute to developing safer and sustainable nanotechnologies.

## Glossary

AChE: Acetylcholinesterase
Ag^+^: Silver ion
AgNO3: Silver nitrate
AgNPs: Silver nanoparticles
AgNW: Silver nanowire: Ag nanowire
AgNW: Silver nanowire: Ag nanowire
AgPL: Ag nanoplate
ASTM: American Society for Testing and Materials
BAF: Bioaccumulation factor
bPEI: Branched polyethyleneimine
CARS: Coherent Anti-stokes Raman Scattering
CAT: Catalase
Cit: Citrate
EC50: Effect concentration causing effect in 50% of exposed organisms
Eco-corona: Environmental corona known
Eco-design: Environmental safety design
ENM: Engineered nanomaterials
ENM/Ps: Engineered nanomaterials and nanoparticles
EPS: Extracellular polymeric substance
GR: Glutathione reductase
GSH: Glutathione
GST: Glutathione transferase
HA: Humic acid
LC50: Lethal Concentration
LCs: Liquid crystals
LPO: Lipid peroxidation
MoA: Mode of action
MXR: MultiXenobiotic Resistance
Nano-bio-interactions: Nanoparticle biological interactions
NEPs: Nano-enabled products
NM: Nanomaterial
NOM: Natural organic matter
NPs: Nanoparticles
PAAm: Poly (allylamine)
PEC: Predicted environmental concentrations
PEG: Polyethylene glycol
PEI: Polyethyleneimine
PLFA: Pony Lake Fulvic Acid
PVA: Polyvinyl alcohol
PVP: Polyvinylpyrrolidone
ROS: Reactive oxygen species
Si: Silicate
SOD: Superoxide dismutase
SRHA: Suwannee River humic acid
SSbD: Safe and Sustainable-by-Design
Tan: Tannic acid
TEM: Transmission electron microscopy
WWTP: Wastewaters from treatment plants
